# Targeting bromodomain-containing proteins: research advances of drug discovery

**DOI:** 10.1186/s43556-023-00127-1

**Published:** 2023-05-05

**Authors:** Zhaoping Pan, Yuxi Zhao, Xiaoyun Wang, Xin Xie, Mingxia Liu, Kaiyao Zhang, Lian Wang, Ding Bai, Leonard J. Foster, Rui Shu, Gu He

**Affiliations:** 1 Department of Dermatology & Venerology and State Key Laboratory of Biotherapy, West China Hospital, Sichuan University, Chengdu, Sichuan 610041 China; 2 Laboratory of Dermatology, Clinical Institute of Inflammation and Immunology (CIII), Frontiers Science Center for Disease-Related Molecular Network, West China Hospital, Sichuan University, Chengdu, 610041 China; 3 State Key Laboratory of Oral Diseases, National Clinical Research Center for Oral Disease, Department of Orthodontics and Pediatrics, West China Hospital of Stomatology, Sichuan University, Chengdu, 610041 China; 4 College of Medical Technology and School of Pharmacy, Chengdu University of Traditional Chinese Medicine, Chengdu, 611137 China; 5 Michael Smith Laboratories, University of British Columbia, Vancouver, BC V6T 1Z4 Canada

**Keywords:** Bromodomain, BD-containing proteins, Drug discovery, Advances

## Abstract

Bromodomain (BD) is an evolutionarily conserved protein module found in 46 different BD-containing proteins (BCPs). BD acts as a specific reader for acetylated lysine residues (KAc) and serves an essential role in transcriptional regulation, chromatin remodeling, DNA damage repair, and cell proliferation. On the other hand, BCPs have been shown to be involved in the pathogenesis of a variety of diseases, including cancers, inflammation, cardiovascular diseases, and viral infections. Over the past decade, researchers have brought new therapeutic strategies to relevant diseases by inhibiting the activity or downregulating the expression of BCPs to interfere with the transcription of pathogenic genes. An increasing number of potent inhibitors and degraders of BCPs have been developed, some of which are already in clinical trials. In this paper, we provide a comprehensive review of recent advances in the study of drugs that inhibit or down-regulate BCPs, focusing on the development history, molecular structure, biological activity, interaction with BCPs and therapeutic potentials of these drugs. In addition, we discuss current challenges, issues to be addressed and future research directions for the development of BCPs inhibitors. Lessons learned from the successful or unsuccessful development experiences of these inhibitors or degraders will facilitate the further development of efficient, selective and less toxic inhibitors of BCPs and eventually achieve drug application in the clinic.

**Supplementary Information:**

The online version contains supplementary material available at 10.1186/s43556-023-00127-1.

## Introduction

**Fig. 1:**
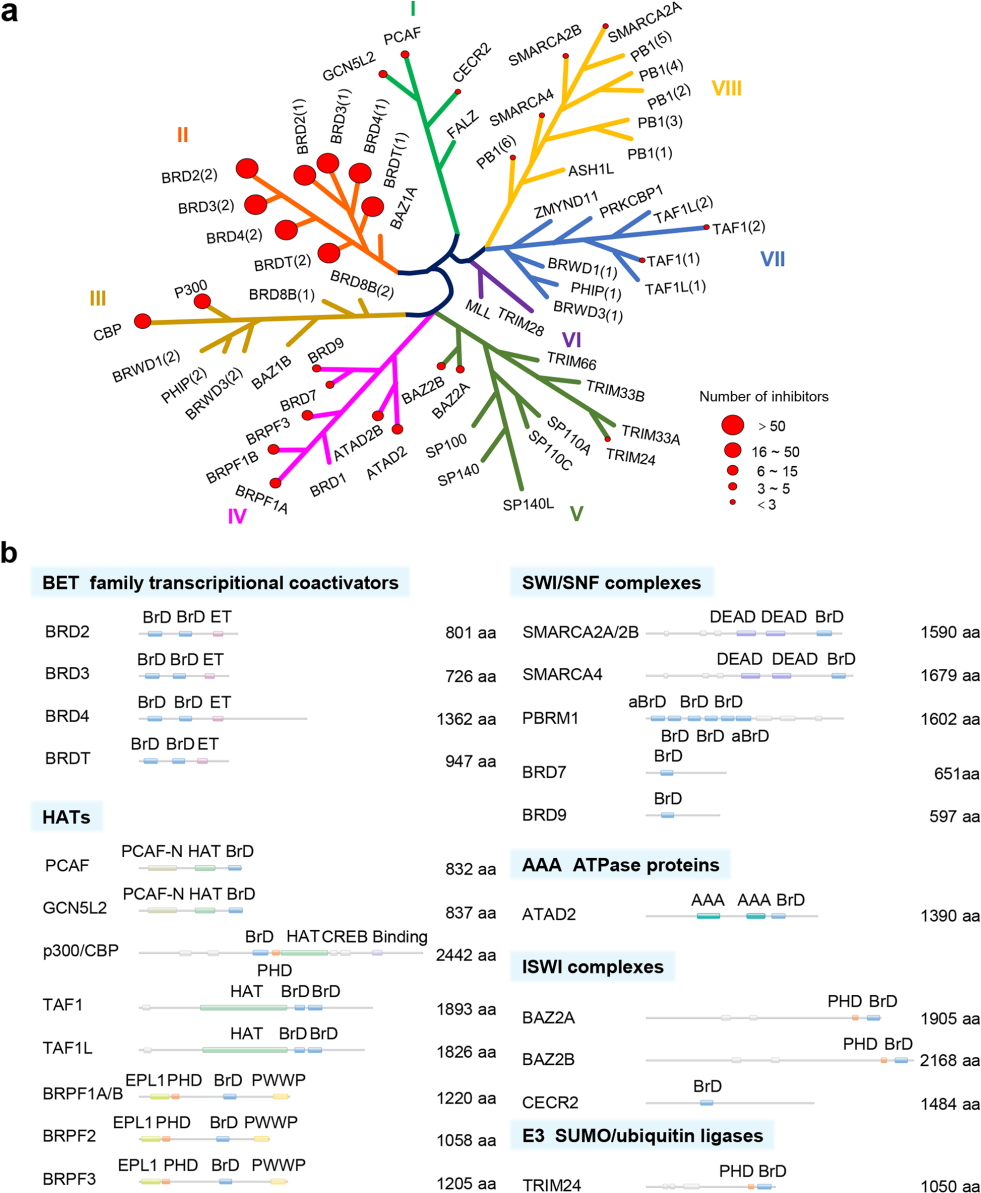
Classification of BD and BCPs. **a** Phylogenetic tree of the human BD family. Eight subgroups are shown in different colors and numbered with roman numerals. The number of inhibitors reported is indicated by the different sizes of red dots. **b** Domain organization of representative BCPs. The major functions of BCPs are bolded and BD is shown in light blue

Bromodomains (BDs) are evolutionarily conserved protein modules consisting of ~ 110 amino acids each that exclusively recognize acetylated lysine (KAc) residues in histones and other proteins. These modules work as pivotal epigenetic mark “readers” [1, 2]. BDs are ubiquitous in eukaryotes with 61 types in humans, and are contained in 46 different BD-containing proteins (BCPs) that can be classified into eight subtypes (Fig. 1a). Usually, BCPs are divided into bromodomain and extra-terminal (BET) and non-BET families according to the number of BDs, domain architecture and homology (Fig. 1b). More specifically, the BET family comprises two BDs, while the non-BET member contains one to six conventional or atypical BDs. The BET family in mammals comprises BRD2, BRD3, BRD4, and the testis-specific BRDT [3–5], while the non-BET family includes histone acetyl-transferases (HATs, such as p300/CBP-associated factor (PCAF), general control of amino acid synthesis protein 5-like 2 (GCN5L2), and E1A-binding protein p300 (EP300, p300)) [6–9], histone methyl-transferases (such as ash1 (absent, small,or homeotic)-like (ASH1L), myeloid/lymphoid or mixed lineage leukemia protein (MLL)) [10, 11], ATP-dependent chromatin remodeling complexes (such as bromodomain adjacent to zinc finger domain 1B (BAZ1B)) [12], helicases (such as SWI/SNF-related matrix-associated actin-dependent regulator of chromatin a2(SMARCA2)) [13], transcriptional coactivators (such as tripartite motif-containing proteins (TRIMs)) [14], nuclear-scaffolding proteins (such as polybromo 1(PB1)) [15], among others.

**Fig. 2:**
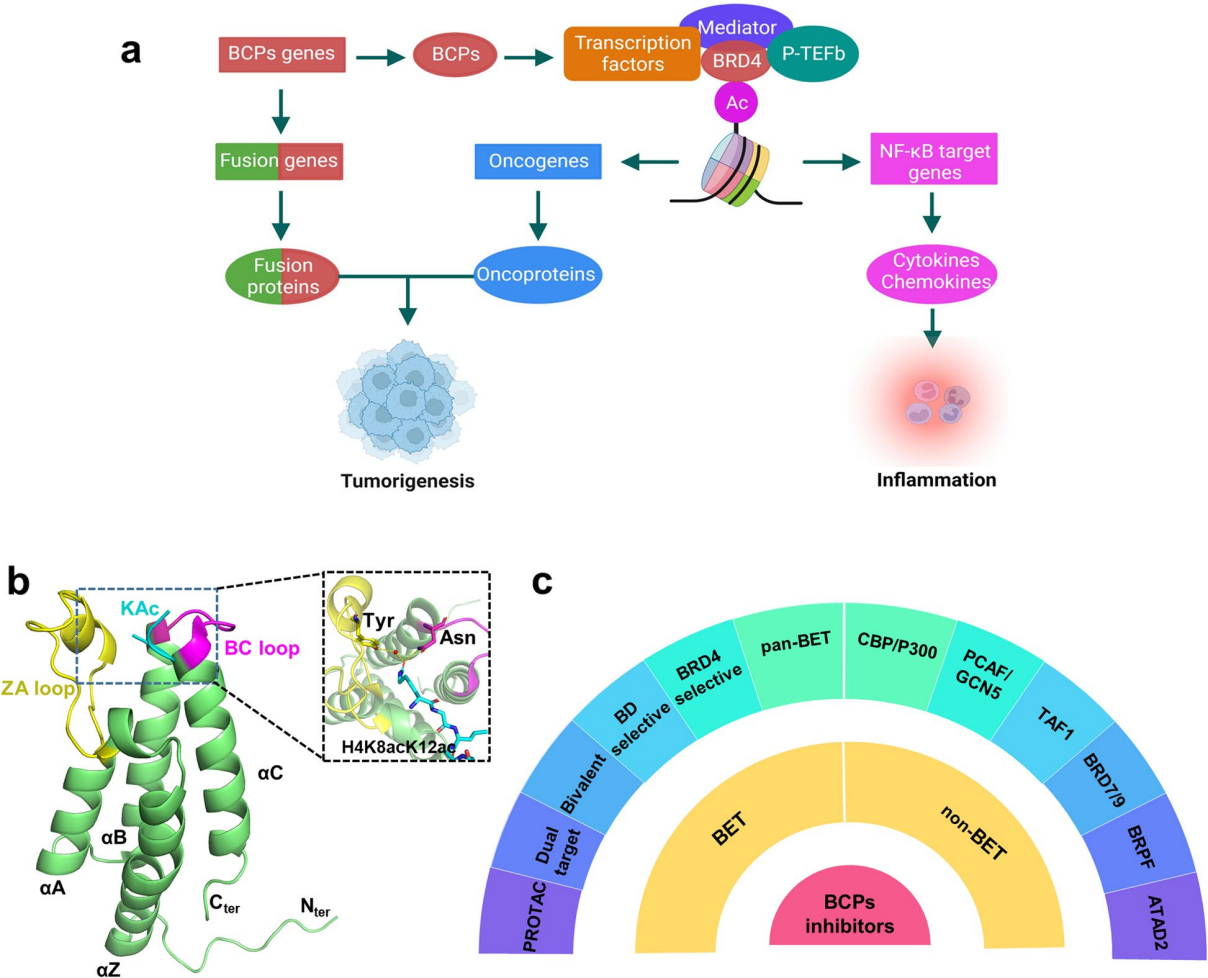
The relationship between BCPs and disease, BD structure, and inhibitor classification.** a** Association of BCPs with the development of diseases (cancer and inflammation). **b** The 3D structure of BRD4 BD1 and the details of the interactions of pocket with an acetylated histone peptide ligand (H4K8acK12ac) in the recognition. The complex structure is derived from RCSB PDB: 3UW9. **c** BCPs inhibitors can be divided into two categories: BET and non-BET inhibitors, with BET inhibitors further subdivided into pan-BET, BRD4-selective, BD1/BD2-selective, bivalent, dual-target inhibitors and PROTACs, and the reported non-BET inhibitors further subdivided into CBP/p300, PCAF/GCN5, TAF1, BRD7/9, BRPF and ATAD2 inhibitors

Structurally, Filippakopoulos et al. uncovered the comprehensive structural features of human BCPs and identified the KAc-specific recognition sites of these proteins, using 33 crystal structures and 4 nuclear magnetic resonance (NMR) models [16]. Despite considerable variation in sequence, BDs share a relatively conserved fold comprising four left-handed anti-parallel α helices (αZ, αA, αB, αC) linked by two hydrophobic loop regions (ZA and BC loops). As expected, these components constitute the pocket responsible for recognizing histone acetylation motifs. Co-crystal structures data indicate KAc is recognized by a central hydrophobic cavity, in which KAc is immobilized to an asparagine residue upon hydrogen bonding [16] (Fig. 2b). Similarly, the vast majority of small molecule BDs inhibitors bind directly to the protein module by forming hydrogen bonds with the conserved asparagine residue, mimicking the binding mode of KAc. Examples of small molecule BDs inhibitors include ( +)-JQ1, I-BET762, I-BET151 and RVX-208 [17–20]. In parallel, non-KAc mimetic molecules can bind the module without forming a canonical hydrogen bond with the asparagine residue – for instance MS7972 and ZL0590, which are relatively weak inhibitors.

Biologically, quite a few evidences support that BCPs are involved with/in transcriptional regulation, chromatin remodeling, DNA damage repair, cell cycle regulation, cellular proliferation, oncogenesis, and inflammatory. Broad biological functions suggest that targeting these proteins may be an effective therapeutic strategy for cancer, inflammation, and the central nervous system (CNS) diseases [5, 21–23]. In a variety of types of malignant tumors, there is a consistent over-expression of multiple BCPs genes on which tumors depend, including *BRD2*, *BRD4*, *ATAD2*, *KAT2A*, etc. [24–26]. The above genes have been identified as oncogenes, and their over-expression is highly correlated with aggressive cancer progression and poor prognosis. BRD4 is necessary for the proliferation and survival of various tumors (ovarian cancer, leukemia, multiple myeloma). It interacts with the positive transcription elongation factor b (P-TEFb) complex composed of cyclinT1/cyclin dependent kinase 9 (CDK9) heterodimer and recruits transcription factors such as MYC, p53, and TWIST, thereby stimulating RNA polymerase II-dependent transcription and ultimately promoting gene expression and cell cycle progression [27]. BRD4 inhibition is confirmed as an effective therapeutic strategy for the treatment of malignancies characterized by pathological activation of c-MYC [28, 29]. In addition, genes encoding BCPs often undergo chromosomal translocations and generate fusions. For example, *BRD4* fusion with the nuclear protein in testis (*NUT*) gene expresses the BRD4-NUT oncoprotein, which plays an important role in the growth maintenance and differentiation block of the lethal malignancy NUT midline carcinoma (NMC) [30]. Moreover, other fusions involving BCPs genes, such as *EP300* fusion with *ZNF384*, *ASH1L* fusion with *C1ORF61*, *SMARCA4* fusion with *CARM1*, *MLL* fusion with *AF4, AF9, or AF10* are also found in tumors such as hematological malignancies, breast cancers, glioblastomas, driving tumor progression [29]. Furthermore, in the course of past studies on BCPs scientists have found that BCPs are also implicated in inflammatory responses. By recognizing and binding acetylated lysines of RelA (p65), BRD4 not only enhances NF-κB transcriptional activation but also facilitates P-TEFb-dependent transcription of a panel of pro-inflammatory NF-κB target genes. Disruption of the interaction between BRD4 and NF-κB reduces the expression of pro-inflammatory cytokines and chemokines such as interleukin-1β (IL-1β), IL-6, IL-12α, and CXCL9, resulting in anti-inflammatory effects [31, 32]. Similarly, modulation of NF-κB-dominated inflammatory pathways by inhibition of BCPs provides benefits in the CNS diseases, such as reducing infarct volume, promoting recovery of brain function and preventing secondary damage [33] (Fig. 2a). There are robust data linking BCPs to cancer, inflammation and other diseases. Pharmacological inhibition of these proteins has shown promising and effective therapeutic effects in a variety of conditions, particularly in the treatment of cancer and inflammatory diseases. In the past decade, the development of BCPs inhibitors represented by ( +)-JQ1, has achieved milestones [34–37], with numerous inhibitors successfully developed and currently under clinical trials, such as Molibresib, Birabresib, CPI-0610, PLX51107, RVX-208, and CCS1477 (Tab. 1).

**Table 1:** BCPs inhibitors in clinical trials

Drug	Interventions	Phase	Conditions	NCT Number	Status
Molibresib	combination with entinostat	I	lymphoma, solid tumors	NCT03925428	withdrawn
	combination with cisplatin/etoposide	I/II	NUT Carcinoma	NCT04116359	withdrawn
	combination with fulvestrant	I	neoplasms	NCT02964507	completed
	monotherapy	I	NMC	NCT01587703	completed
	crossover assignment (rifampin/itraconazole)	I	neoplasms	NCT02706535	completed
	monotherapy		NMC	NCT03702036	no longer available
	combinationwith androgen deprivation therapy	I	CRPC	NCT03150056	terminated
	combination with trametinib	II	solid tumors	NCT03266159	withdrawn
	monotherapy	II	neoplasms	NCT01943851	completed
Birabresib	monotherapy	I	AML, DLBCL	NCT02698189	terminated-limited efficacy
	monotherapy	I	NMC, TNBC, NSCLC, CRPC	NCT02698176	Terminated-limited efficacy
	monotherapy	I	NMC, TNBC, NSCLC, CRPC	NCT02259114	completed
	monotherapy	I	AML, DLBCL, ALL, MM	NCT01713582	completed
	monotherapy	II	GBM	NCT02296476	terminated
	combination with vidaza	I/II	AML	NCT02303782	withdrawn
CPI-0610	monotherapy	I	MM	NCT02157636	completed
	monotherapy	I	lymphoma	NCT01949883	completed
	monotherapy	I	advanced malignancies	NCT05391022	recruiting
	combination with ruxolitinib	I/II	myelofibrosis	NCT02158858	active, not recruiting
	monotherapy	II	MPNST	NCT02986919	withdrawn
	combination with ruxolitinib	III	myelofibrosis	NCT04603495	recruiting
PLX51107	monotherapy	I/II	GVHD	NCT04910152	recruiting
	combination with azacitidine	I	AML, MDS	NCT04022785	recruiting
	monotherapy	I	advanced malignancies	NCT02683395	terminated
ODM-207	monotherapy	I/II	solid tumors	NCT03035591	completed
**29**	monotherapy	I/II	advanced malignancies	NCT02431260	terminated-PK variability
	monotherapy		metastatic RCC	NCT03896815	no longer available
CC-90010	monotherapy	I	astrocytoma, glioblastoma	NCT04047303	active, not recruiting
	monotherapy	I	NHL, solid tumors	NCT03220347	active, not recruiting
	combination with temozolomide	I	glioblastoma	NCT04324840	recruiting
	combination with BMS-986158	I	pediatric cancer	NCT03936465	recruiting
BMS-986158	monotherapy or combination therapy	I/II	myelofibrosis	NCT04817007	recruiting
	monotherapy or combination therapy	I/II	advanced tumors	NCT02419417	completed
	combination therapy	I/II	MM	NCT05372354	recruiting
ABBV-075	monotherapy or combination with venetoclax	I	cancers	NCT02391480	completed
	monotherapy or combination therapy	I	myelofibrosis	NCT04480086	active, not recruiting
BI 894,999	monotherapy	I	advanced malignancies	NCT02516553	completed
RVX208	monotherapy	I/II	fabry disease	NCT03228940	unknown
	monotherapy	I/II	dyslipidemia, atherosclerosis	NCT00768274	completed
	monotherapy	II	atherosclerosis, CAD	NCT01058018	completed
	monotherapy	II	CAD, dyslipidemia	NCT01423188	completed
	monotherapy	II	diabetes	NCT01728467	completed
	combination with rosuvastatin/atorvastatin	II	dyslipidemia, CAD	NCT01863225	terminated
	combination with atorvastatin/ rosuvastatin	III	diabetes, CAD	NCT02586155	completed
ABBV-744	monotherapy	I	AML	NCT03360006	terminated
	monotherapy or combination therapy	I	myelofibrosis	NCT04454658	recruiting
AZD5153	monotherapy or combination with olaparib	I	solid tumors	NCT03205176	completed
	combination with acalabrutinib	I	NHL	NCT03527147	completed
CCS1477	monotherapy or combination therapy	II	advanced tumours	NCT03568656	recruiting
	monotherapy or combination therapy	I/II	haematological malignancy	NCT04068597	recruiting

Sourced from https://clinicaltrials.gov/ (March 2023)

*Abbreviations* used in the table: *ALL* Acute lymphoblastic leukemia, *AML* Acute myeloid leukemia, *CAD* Coronary artery disease, *CRPC* Castration-resistant prostate cancer, *DLBCL* Diffuse large B-cell lymphoma, *GBM* Glioblastoma multiforme, *GVHD* Graft versus host disease, *MDS* Myelodysplastic syndrome, *MM* Multiple myeloma, *MPNST* Malignant Peripheral Nerve Sheath Tumors, *NHL* Non-Hodgkin’s lymphoma, *NMC* NUT midline Carcinoma, *NSCLC* Non-small cell lung cancer, *RCC* Renal cell carcinoma, *SCLC* Small cell lung cancer, *TNBC* Triple negative breast cancer

The mechanism of action of the currently developed drugs targeting BCPs is mainly to inhibit the interaction of BCPs with KAc and to down-regulate the protein content of BCPs. Therefore, we focused on a detailed review of the research and advance of inhibitors and degraders targeting BCPs. According to the classification of BCPs, we described the BET family and non-BET family drugs one by one in this paper. The review covered the history of drug development, structural features, biological activities, interactions with target proteins and the current status of clinical applications, highlighting the molecular structures and biological activities. We concluded the review by discussing the potential effective measures to develop efficient, selective and less toxic inhibitors of BCPs, suggesting effective strategies to overcome resistance, and describing the current challenges facing inhibitors of BCPs.

## The BCPs inhibitors

Building on the close and rich correlation between BCPs and disease and the proof that interfering with BCPs-KAc binding is an effective therapeutic strategy, there has been a wave of development of BCPs inhibitors in academia and industry with dazzling results [38]. Conventionally, inhibitors can be categorized into two groups: BET inhibitors and non-BET inhibitors (Fig. 2c). Available data suggest that BET inhibitors are well-developed and very diverse, compared to non-BET ones, which are relatively few and still under development. Based on their selectivity and mechanism of action, BET inhibitors can be further subdivided into pan-BET inhibitors, BRD4-selective inhibitors, BD1/BD2-selective inhibitors, bivalent inhibitors, dual-target inhibitors and PROTACs, while non-BET inhibitors can be sub-grouped into CBP/p300, PCAF/GCN5, TAF1, BRD7/9, BRPF and ATAD2 inhibitors. We will discuss specific classes of BET and non-BET inhibitors in the sections below.

## BET inhibitors

### Pan-BET inhibitors

#### Thienotriazolodiazepines or triazolodiazepines

**Fig. 3:**
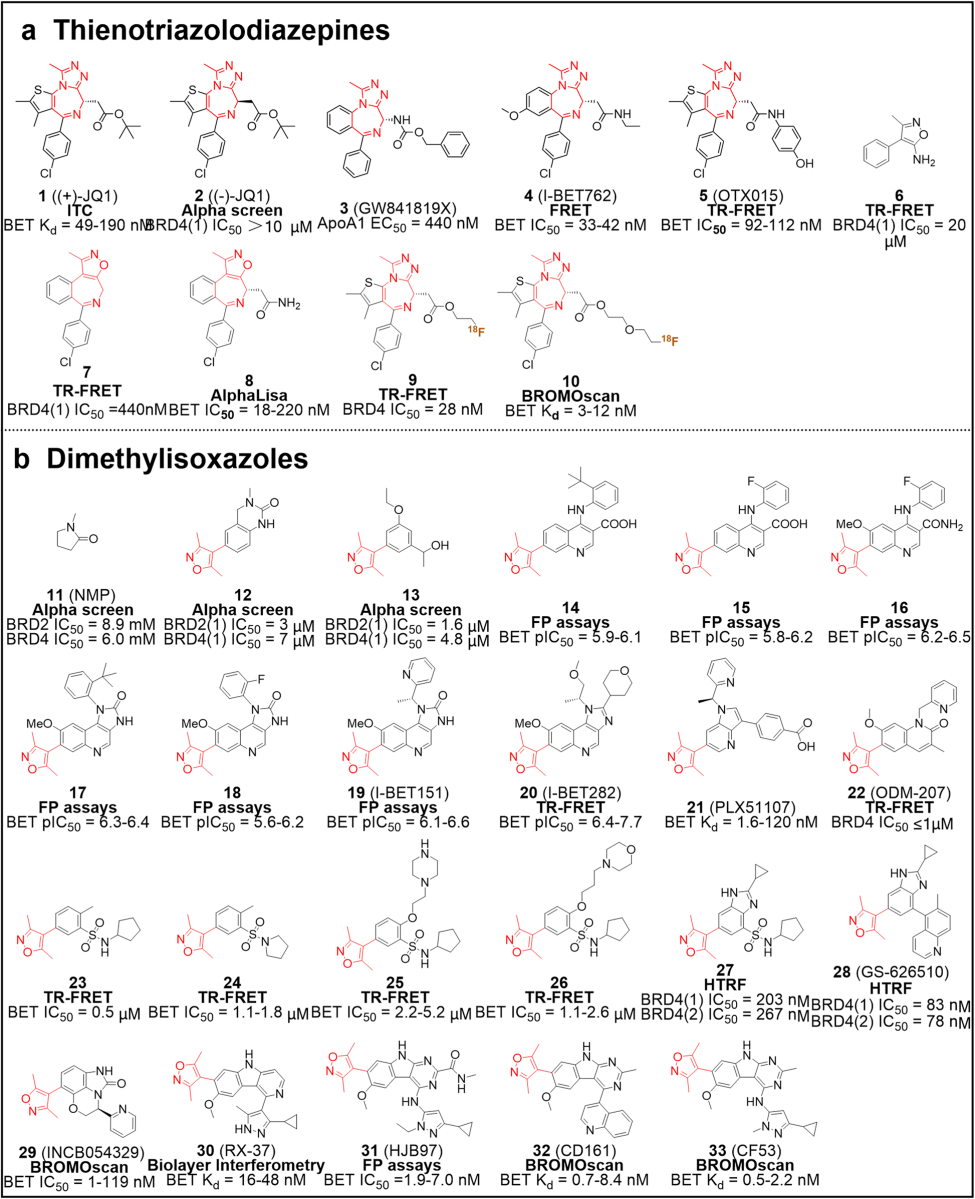
Chemical structures of pan-BET inhibitors. **a** triazolodiazepine pan-BET inhibitors **1** − **10**. **b** Dimethylisoxazole pan-BET inhibitors **11** − **33**. The key scaffolds are highlighted in red and activity test methods are bolded. FRET, fluorescence resonance energy transfer; TR-FRET, time-resolved FRET. FA/FP, fluorescent anisotropy/fluorescence polarization; HTRF, homogeneous time-resolved fluorescence

Back in 1996, the Mitsubishi Tanabe Pharma Corporation of Japan described in a patent a class of thienotriazolodiazepine-based compounds that could be used for the treatment and prevention of inflammatory bowel diseases (IBD, such as ulcerative colitis and Crohn’s disease). These compounds acted on cellular adhesion, and their high safety profile was verified in animal studies [39]. Further elaboration by the company showed that thienotriazolodiazepine compounds inhibited costimulatory signal from CD28 on T cells and had a positive effect in preventing rejection of organ transplantation, autoimmune diseases, and allergic reactions [40]. Notably in the 2009 patent report on anti-tumor agents, compounds containing thienotriazolodiazepine inhibited the binding of acetylated histone to BCPs, effectively shrinking or killing cancer cells in mammals [41]. Benefiting from the long-standing and extensive clinical application basis of anti-anxiety and sedative drugs (e.g., alprazolam and triazolam) containing a benzodiazepine (BZD) skeleton, thienotriazolodiazepines have well-established druggability, including high safety, low toxicity, high bioavailability and synthetic accessibility [42]. Also, the favorable inhibitory activity of thienotriazolodiazepines against BCPs makes them ideal scaffolds for the construction of specific inhibitors. Inspired by the well-characterized pharmacology of these small molecules using the thienotriazolodiazepine as the core scaffold, Filippakopoulos and co-workers synthesized a novel thieno-triazolo-1,4-diazepine ( +)-JQ1 (**1**) equipped with tert-butanol esters in high yields via a seven-step total synthesis [17] (Fig. 3a). As indicated by differential scanning fluorimetry (DSF) test data, compound **1** exhibited highly selective inhibitory activity against the BET family with temperature shifts from 4.2℃ (BRDT(1)) to 10.1 ℃ (BRD4(1)) and K_d_ values from 49 nM (BRD4(1)) to 190 nM (BRDT(1)), while the shifts or K_d_ were hardly detectable for non-BET proteins. However, unlike its enantiomer, (-)-JQ1 (**2**) did not interact with BDs. Further experiments showed that compound **1** could competitively inhibit the binding of Histone H4 peptide characterized by four acetylation modifications to BRD4, reaching an IC_50_ of 33 nM for BD1 and 77 nM for BD2. The co-crystal structure indicated that compound **1** fitted nicely into the KAc pocket of BRD4, while there was a key hydrogen bond between the triazole ring and asparagine (Asn140 in BD1, Asn429 in BD2). In the ZA and BC loops, the formation of hydrophobic interactions with conserved residues also serves to stabilize the complex (Supplementary Fig. 1a). On the other hand, the collision of compound **2** with residues Leu92 and Leu94 in the spatial structure provides an explanation for its lack of biological activity. Displacing BRD4 from nuclear chromatin, compound **1** was shown to effectively cause nuclear protein in testis (NUT) midline carcinoma (NMC) regression and improve the overall survival, accompanied by induction of squamous differentiation, proliferation inhibition, and apoptosis activation. In multiple myeloma (MM), compound **1** could effectively down-regulate *MYC* transcription due to its BET inhibitory activity, contributing to an anti-proliferative effect in a time- and dose-dependent manner. As a first-in-class selective BET inhibitor, compound **1** is currently employed in therapeutic research for various diseases, including cancers, inflammation diseases, diabetes, and HIV infection [43–47]. Unfortunately, due to its short half-life, compound **1** is only widely used as a tool drug or chemical probe.

Meanwhile, another triazolodiazepine I-BET762 (molibresib, I-BET or GSK525762A, compound **4**), was developed by the Medicines Research Centre of GlaxoSmithKline (GSK), as a potent BET inhibitor (Fig. 3a). Compound **4** is derived from the BZD GW841819X (**3**), which was originally used as an apolipoprotein A1 (ApoA1) enhancer (EC_50_ = 440 nM) [48]. Screened from a structure–activity relationship (SAR) optimization aiming at improving the physicochemical properties and pharmacokinetic (PK) parameters, compound **4** shows high affinities toward different BET proteins, thus being considered a pan-BET inhibitor [49]. As a highly selective BET inhibitor, compound **4** not only has no inhibition towards non-BET family, but also has no binding activity to non-BCPs. Similar to compound **1**, compound **4** is located in the KAc recognition pocket and its triazole moiety forms hydrogen bonds with the conserved Asn140 and Tyr97 (Supplementary Fig. 1b). Data from the literature demonstrates that compound **4** has a high affinity (K_d_ = 50.5 − 61.3 nM) and potent inhibitory capacity (IC_50_ = 32.5 − 42.5 nM) for BET. This compound can selectively down-regulate a variety of lipopolysaccharide (LPS)-induced inflammatory genes and suppress the expression of IL-6, IL-1β, IL-12α, interferon beta 1 (IFN-β1) and chemokine (C-X-C motif) ligand 9 (CXCL9), which are pivotal cytokines and chemokines in the inflammatory response [18, 49]. In subsequent reports, inhibitor **4** has been utilized as an anti-tumor compound, especially in hematological malignancies and NMC. Indeed, several clinical trials (mainly phase I-II) have been performed to ascertain toxicity, and ideal dose while an expansion study has investigated the safety, PK/pharmacodynamics (PD), and clinical therapeutic effect of compound **4** in subjects with hematological malignancies and solid tumors. The successful development of the first two triazolodiazepine inhibitors has inspired scientists to identify other BET inhibitors, resulting in numerous novel molecules have been developed successively.

Birabresib (also called OTX015 or MK8628, compound **5**), is derived from the replacement of the tert-butanol group by the 4-hydroxyphenyl group at the C6 of compound **1** (Fig. 3a). Compound **5** retains the triazolodiazepine as its core skeleton. It was initially discovered in a screening for anti-cell adhesion activity and subsequently used as an inhibitor of BET binding to acetylated histones (IC_50_ = 92 − 112 nM), capable of potent anti-tumor proliferative ability with a GI_50_ of 60 nM to 200 nM for hematological malignancies. Compound **5** shows remarkable inhibitory activity against BET in vitro, and excellent anti-tumor activity against a variety of solid tumors and hematological malignancies in vivo*,* including breast cancer, nasopharyngeal carcinoma, lung cancer, NMC, glioblastoma, neuroblastoma, acute myeloid leukaemia, and lymphoma [50–52]. Multiple Phase I clinical trials were conducted to investigate the safety, efficacy, and recommended dose of compound **5**. These studies have shown that the most common side effects are fatigue, gastrointestinal disorders, hyperbilirubinemia and reversible thrombocytopenia [53, 54].

Emerging evidence suggests that BET inhibitors can effectively regulate the expression of disease-associated genes, including *MYC*, *BCL2* and *NFκB*, by interfering with the interaction between BET and chromatin acetyl-lysine tails. Constellation Pharmaceuticals described a novel selective BET inhibitor CPI-0610 (**8**) with isoxazoloazepin (regarded as an atypical triazolodiazepine) as the scaffold (Fig. 3a). In their early research, the active fragment aminoisoxazole **6** (IC_50_ = 20 μM) was obtained by DSF screening and was shown to form two hydrogen bonds with Asn140 and one water-mediated hydrogen bond with Tyr97 [55]. To fill the hydrophobic region of the binding pocket to optimize compound activity, a range of compounds was then prepared by fusing fragment **6** with a seven-membered heterocycle (**7**). A systematic SAR study showed that the optimal compound **8** exhibited considerable BET inhibitory activity (TR-FRET, IC_50_ = 40 nM for BRD4; AlphaLisa Assay, IC_50_ = 18 − 220 nM for BET) and metabolic stability, illustrating that combined hydrogen bonding and hydrophobic interactions are critical for activity optimization in such compounds [56]. Compound **8** was identified as a candidate for clinical trials due to its ability to suppress tumor growth and down-regulate MYC substantially in vivo, as well as its acceptable drug toxicity [56, 57]. Several clinical trials were conducted to establish its efficacy, safety and toxicity profile in patients with lymphoma, MM, myelofibrosis, and advanced malignancies [58].

Given the emerging attention on the role of BET in the central nervous system (NCS) pathology, BET inhibitors that can cross the blood–brain barrier (BBB) are of special interest [59] (Fig. 3a). Changning Wang et al. introduced different lengths of fluorinated alkyl and fluorinated polyethylene glycol side chains into compound** 1** at the C6 position to prepare a series of novel BET inhibitors displaying moderate to potent inhibitory activity with an IC_50_ of 0.028 to 50.2 μM, that might be used as potential positron emission tomography (PET) radiotracer [60]. Among them, compounds** 9** and **10** were selected as candidates for further studies. Molecular docking indicates that both can occupy the KAc binding pocket, while 4-chlorophenyl forms hydrophobic interactions with the “WPF shelf” (Trp81, Pro82, and Phe83) and the fluor substituted side chain forms hydrogen bonds with Asn140 and Tyr97 deep inside the pocket. Compound **10** was shown to exhibit a highly selective binding activity to BET family proteins, a reasonable BBB penetration and metabolic stability. After being labeled with ^18^F, PET evaluation shows that** [**^**18**^**F] 9** and **[**^**18**^**F] 10** are able to effectively penetrate the BBB with a maximum standardized uptake values of 1.7 and 2, respectively. Therefore, this study provides an exploitable tool (**[**^**18**^**F] 10**) to investigate the distribution of BET in the brain and unveils new strategies for the development of PET radiotracer.

#### Dimethylisoxazoles

In 2011, Heightman et al. found that *N*-methyl pyrrolidinone (NMP, **11**), a colorless transparent oily solvent, demonstrated weak inhibitory activity against BRD2 and BRD4 with an IC_50_ of 8.9 and 6.0 mM respectively [61] (Fig. 3b). Further activity studies revealed that commercially available compound **12** exhibited a relatively stronger potency against BRD2(1) (IC_50_ ≈3 μM) and BRD4(1) (IC_50_ ≈7 μM). Determination of its co-crystal structure confirmed that its dimethylisoxazole fragment mimicked KAc and lay in the binding pocket rather than the quinazolinone [62]. Considerable effort was devoted to the mono- or di-substituted modification of 5-dimethyl-4-phenylisoxazole scaffolds to overcome its susceptibility to oxidation and to explore the interaction with the binding pocket. Among these molecules, compound **13** showed considerable selectivity for BET over non-BET proteins (CREBBP, PCAF, and PB1) and inhibitory activity against BRD2(1) and BRD4(1) with an IC_50_ of 1.6 and 4.8 μM respectively. Contemporary, GSK identified a second class of ApoA1 up-regulators (compounds **14** and **15**) characterized by a 3,5-dimethylisoxazole (or isoxazoloquinoline) that bound strongly to BDs. Replacing the acid at the 3-position of quinoline with a carboxamide group and introducing methoxy at the 6-position (**16**) improved the physicochemical properties and BET inhibitory activity of these compounds. Cyclization of the 4-position secondary amine with the 3-position carboxamide to obtain azolidinones imposed restrictions on rotation, thereby weakening the inhibition of CYP450 by these compounds (**17 and 18**) [63, 64]. The introduction of pyridin-2-ylmethyl (IBET-151, or GSK1210151A, **19**) led to a reduced CYP liability and higher potency. In preclinical models, compound **19** could reduce the production of pro-inflammatory cytokine IL-6 induced by LPS, avoiding LPS-induced mortality in mice. Evaluation of the effectiveness of the treatment showed that compound **19** has excellent efficacy against a panel of leukemic cell lines bearing diverse MLL fusions [20]. Mechanistically, compound **19** was shown to promote cell cycle arrest and apoptosis through displacing BRD3/4, polymerase-associated factor complex (PAFc), and super elongation complex (SEC) from chromatin, culminating in the suppression of important oncogenes (including *BCL2*, *MYC* and *CDK6*). Indeed, **19** exhibits promising therapeutic effects in mouse models of leukemia. Overall, this indicates that displacing bromodomian proteins from chromatin may be a valuable epigenetic treatment strategy.

Since the activity of **19** in whole blood assays was not very gratifying, different structural modification strategies were used to enhance the non-cellular potency or reduce the bias between cellular and non-cellular assays in vitro*,* as well as to lower its CYP activity. GSK reported a new molecule derived from **19** by replacement of the pyridine with ether at the imidazoquinolinone 1-position and introducing a 4-tetrahydropyranyl at the 2-position, namely I-BET282 (**20**) [65] (Fig. 3b). As a pan-BET inhibitor, compound **20** can selectively bind to all BET bromodomains with high affinity (pIC_50_ = 6.4 − 7.7 and K_d_ = 8.1 − 140 nM). The nitrogen and oxygen atoms of indimethylisoxazole respectively bind to the Tyr97 and Asn140 in the pocket, through a conventional or water-mediated hydrogen bond. The branched-chain methoxypropan-2-yl can bind into the “WPF shelf” region and form a hydrophobic interaction with Leu92, while the tetrahydro-2*H*-pyran withdraws from the channel formed by Trp81 and Leu92 (Supplementary Fig. 1c). As a result, compound **20** and the bromodomain of BRD4 complement each other perfectly. In preclinical tests using three different animal models, compound **20** demonstrates moderate to low clearance and acceptable oral bioavailability, as well as I-BET282E (the mesylate salt form of **20**) shows significant arthritis therapeutic efficacy in Wistar rats. Although I-BET282E progressed into phase I clinical trial to evaluate its safety, PK/PD, and preliminary clinical activity in subjects with advanced or recurrent solid tumors (NCT02630251), the trial was ultimately terminated in 2017 due to the development of I-BET762(compound **4**) and a better understanding of its risk–benefit profile.

PLX51107 (**21**) is characterized by a dimethylisoxazole but with distinct structural skeleton (Fig. 3b). It is obtained through a scaffold-based and crystallography-guided design strategy [66]. Differently from other BET inhibitors, in addition to forming hydrophobic interactions with the protein, the benzoic acid in the structure of **21** also forms a salt bridge with Lys91 while crossing the ZA loop. The identification of salt bridges suggests that additional binding may be allowed in the ZA loop to promote shape complementation between the inhibitor and BRD4. Compound **21** displays nanomolar binding potency for BET family with a K_d_ of 1.6 − 120 nM and shows a preference for BD1 over BD2. Compound **21** has a significant anti-proliferative activity in primary Chronic lymphocytic leukemia (CLL) cells stimulated by CpG in a dose-dependent manner. In distinct in vivo models, **21** exhibits significant anti-hematological tumor activity, notably reducing leukemia cells and spleen size. A preliminary clinical phase Ib/IIa trial in eight patients with refractory and relapsed malignancies show a stable disease after administration of compound **21** (NCT04022785). Common adverse reactions include fatigue and gastrointestinal reactions, and increases in bilirubin and INR. Another clinical trials using PLX51107 are currently recruiting volunteers to evaluate its efficacy in patients with steroid-refractory graft versus host disease (NCT04910152).

Structurally related to **19**, ODM-207 **(22)** is also a potent pan-BET inhibitor (Fig. 3b). Multiple preclinical animal studies have shown that **22** exerts anti-cancer activity against prostate cancer, leukemia, and breast cancer. The results of a recent phase I trial of compound **22** shows that although the dose increase to 2 mg/kg is safe, the therapeutic window is narrow [67]. The main adverse reactions are thrombocytopenia and digestive tract discomfort, while platelet counts usually recover after cessation. GSK developed a new series of phenylisoxazole sulfonamides BET inhibitors via fragment-based drug discovery (FBDD) [68]. Compounds **23** and **24** exhibit sub-micromolar to micromolar levels of BET inhibitory activity, but unfortunately with the fatal drawback of poor solubility (< 1 or 5 μg/ml when pH = 7). To improve solubility, compounds **25** and **26** were yielded by removing the methyl group at the *o*-position of the sulfonamide on the phenyl ring and introducing polar branched chains (Fig. 3b). As expected, the solubility was greatly enhanced (540 and 210 μg/ml for **25** and **26** respectively) by these structural modifications, and there was only a relatively slight loss of activity. Later, Breckenridge et al. developed a cyclopropyl benzimidazole **27** on the basis of compound **23** via lead optimization, which had superior cell potency and protein inhibition [69]. Further optimization to obtain GS-626510 (**28**) with an increased inhibitory activity against BET, higher permeability, and stability.

Since the conserved dimethylisoxazole can effectively maintain the interaction with BD to ensure pharmacological activity, this opens up the possibility of molecular diversity. INCB054329 (**29**) shows inhibitory activity at low nanomolar concentrations against the binding of BET to acetylated histone H4. In cellular assays **29** inhibits the expression of c-MYC, the growth of lymphoma, myeloma, and AML cell lines, and sensitizes cells to the growth inhibition, DNA damage, and apoptosis induced by olaparib. Oral administration of **29** is also effective in inhibiting tumor growth in various in vivo hematological tumor models. Because of the short half-life and high PK variability in the phase I trial, the evaluation of **29** was terminated. Perhaps additional follow-up dose optimization of compound **29** and improved screening strategies can benefit patients [70].

Wang et al. reported a series of new BET inhibitors containing γ-carboline (Fig. 3b). By analyzing the co-crystal structure of compound **19** and BRD4(1), they developed a new γ-carboline-containing BET inhibitor RX-37 (**30**), with Ki ranging from 3.2 to 24.7 nM and high selectivity for BET [71]. After further optimization, HJB97 (**31**) with the same scaffold was obtained, which exhibited a favorable binding affinity to BRD2, BRD3, and BRD4 (Ki < 1 nM), superior to that of compounds **1** and **5**. Later, compound **31** was implemented in the design of PROTACs for targeted degradation of BET [72]. To solve the problem that compound **30** was difficult to synthesize on a large scale, an alternative tricyclic skeleton was employed to CD161 (**32**) [73]. With excellent growth inhibition in AL and breast cancer cell lines, compound **32** has also been confirmed as an orally available candidate demonstrating exceptional PK profile and tumor suppression ability in vivo. Furthermore, in an attempt to obtain a single crystal structure, they removed the restricted rotation via structural modification, resulting in the acquisition of compound CF53 (**33**). Compound **33** effectively inhibited the growth of triple-negative breast carcinoma and AL cells, and was identified as a potent, orally available, and clinically valuable drug candidate [74].

#### Tetrahydroquinolines

**Fig. 4:**
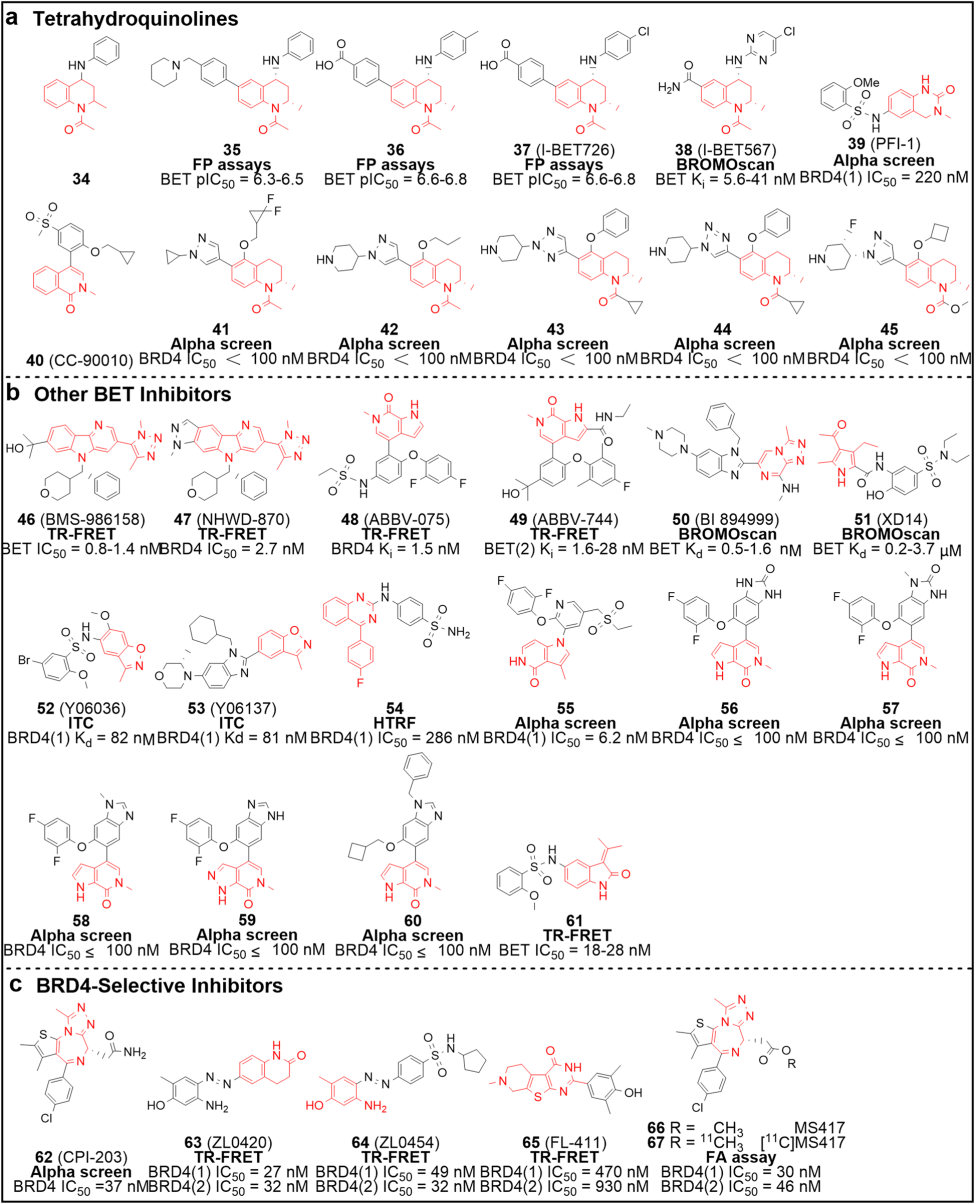
Chemical structures of pan-BET and BRD4-selective inhibitors. **a** THQ pan-BET inhibitors **34** − **45**. **b** other BET inhibitors **46** − **61**. **c** BRD4-selective inhibitors **62** − **67**. The key scaffolds are highlighted in red and activity test methods are bolded. ITC, isothermal titration calorimetry

The tetrahydroquinoline **(THQ)** scaffold is frequently associated with drug discovery, GSK employed it to assemble a series of ApoA1 up-regulators that were subsequently revealed to function as potent BET inhibitors [75] (Fig. 4a). Initially, compound **34** was picked out by high-throughput screening (HTS) and was shown capable of up-regulating ApoA1 several-fold at 10 μM. The optimization of the molecule was carried out by introducing *p*-(piperidin-1-methyl)phenyl at the 6-position to obtain compound **35**, which showed significantly improved activity and solubility. In addition to being an ApoA1 activator, compound **35** is also a potent BET inhibitor with an average pIC_50_ of 6.4. However, compound **35** exhibits moderate inhibitory activity against each CYP450 isoforms with a minimum IC_50_ value of only 3 μM. Therefore, investigators performed additional structural optimization to attenuate its effect on CYP450 and yielded compounds **36** and I-BET726 (**37**). Since the two shared very similar properties, only compound **37** was selected for further studies. In subsequent binding and selectivity tests, compound **37** showed high activity and specificity against the BET family proteins. The *N*1-acetyl group of the inhibitor was shown to form a hydrogen bond with the conserved Asn140 in BRD4 (Asn156 in BRD2) (Supplementary Fig. 1d). Importantly, compound **37** no longer exhibited the worrisome CYP450 inhibitory potency (IC_50_ > 10 μM). Moreover, **37** exhibited favourable PK properties in different preclinical models, including low to moderate clearance, apparent volume of distribution, bioavailability, and long half-life. In the LPS-stimulated sepsis model, compound **37** was shown effective in improving the survival rate of mice after intravenous injection. Down-regulation of *MYCN* and *BCL2* expression suggests that **37** is also a potential BET inhibitor relevant for neuroblastoma treatment [76]. Via Free-Wilson analysis, GSK identified a novel THQ molecule I-BET567 (**38**), with a bias to bind eight bromodomains of BET relative to non-BET proteins (Ki = 5.6 − 41 nM). Due to its improved solubility (> 600 μg/mL), permeability, PK, and efficacy, the orally administrable compound **38** is an ideal candidate worthy of further investigation [77].

PFI-1 (**39**) is derived from the 3,4-dihydro-3-methyl-2(1*H*)-quinazolinone fragment and achieves a good balance between pharmacological activity and lipophilicity [78] (Fig. 4a). As a selective chemical probe, compound **39** shows a preference for BET family proteins, with *T*_*m*_ shifts of 2.1 − 6.5 °C. It moderately inhibits IL-6 production induced by LPS (EC_50_ = 1.89 μM). Another orally available, and reversible BET inhibitor, CC-90010 (**40**), was developed in recent years to inhibit tumor growth either alone or in combination (specific data not published). Phase I clinical trial results show that compound **40**, when administrated alone, shows favorable anti-tumor effects in patients with advanced solid tumors or refractory/relapsed non-Hodgkin lymphoma [79, 80]. Compounds **41** − **45** are BET inhibitors with the THQ template disclosed in the patent literature, while only the inhibitory activity against BRD4 (BD1 IC_50_ < 0.1 μM) is documented [81].

#### Other pan-BET inhibitors

Other structurally diverse BET inhibitors come from Bristol-Myers Squibb (BMS), Yale Cancer Center, AbbVie, and Boehringer-Ingelheim show promising pharmacologic activity (Fig. 4b). Both BMS-986158 (**46**) and NHWD-870 (**47**) are BET inhibitors structurally characterized by a triazole coupling tricyclic with strong anti-tumor effects in NCI-H211 and MDA-MB231 cell lines [82, 83]. The respective IC_50_ of **46** in NCI-H211 and MDA-MB231 are 6.6 nM and 5 nM, while those of **47** are 2 nM and 1.6 nM. ABBV-075 (**48**), a selective BET inhibitor developed by AbbVie, has progressed to phase I clinical trials and demonstrated superior anti-tumor activity in solid tumors and hematological malignancies through multiple mechanisms, including blocking G1 phase, promoting apoptosis, and disrupting the tumor micro-environment [84]. Further optimization resulted in ABBV-774 (**49**) which shows a significant preference for BD2 and a greater than 290-fold selectivity over BD1. Compound **49** displays potent activity in prostate cancer models, with relatively mild thrombocytopenia and gastrointestinal toxicity. These notable optimizations corroborate that the development of BD2-selective inhibitors may be a feasible strategy to develop safe and effective anti-cancer drugs [85, 86]. BI 894,999 (**50**)**,** a member of triazolopyrazines disclosed by Boehringer-Ingelheim, is a highly selective BET inhibitor with a *K*_*d*_ of 0.49 − 1.6 nM. Monotherapy or combination with other drugs showed that compound **50** exerts promising anti-AML efficacy in vitro and in vivo [87, 88]. Professor Günther employed 4-acyl pyrroles to mimic the KAc and screened XD14 (**51**) as a candidate inhibitor [89]. Compound **51** can selectively bind to BET at submicromolar levels (except BRDT) and inhibit the growth of leukemia cells. Y06036 (**52**) and Y06137 (**53**), structurally characterized by benzo[*d*]isoxazole, exhibit potent binding activity to BET proteins (for BRD4, *K*_*d*_ = 82 nM and 81 nM respectively) and high selectivity over non-BET ones [90]. As new chemotype lead compounds, they effectively inhibit the growth of prostate cancer cells with the expression AR and MYC being suppressed, and show excellent therapeutic activity in castration-resistant prostate cancer (CRPC) mouse xenograft models. Very recently, the 4-phenylquinazoline **54** was described as a potential candidate for the treatment of cardiac fibrosis [91]. Moreover, several inhibitors with distinct structures (**55** − **61**) and IC_50_ values at the nanomolar level have also been reported in patents, showing potential anti-proliferative activity against a variety of cancer cells [92].

### BRD4-selective inhibitors

Among BET family proteins, BRD4 is the most studied subtype and a promising drug target. However, the lack of selectivity of most pan-BET inhibitors for individual BET members has led to ambiguity in the perception of the exact mechanism of action of these chemicals and unanticipated adverse drug reactions. Therefore, the development of BRD4-selective inhibitors is urgently needed (Fig. 4c). CPI-203 (**62**), structurally featured by triazolodiazepine, exhibits favorable inhibitory activity against BRD4, MYC, and IL-6 with IC_50_ values of 37 nM, 99 nM, and 30 nM, respectively. Combined with lenalidomide, compound **62** demonstrated synergistic anti-tumor effects in inducing cell death and apoptosis, which may benefit patients with mantle cell lymphoma (MCL) resistant to proteasome inhibitors [93, 94]. Through structure-based drug design, Zhou et al. discovered two selective BRD4 inhibitors, namely ZL0420 (**63**) and ZL0454 (**64**). Both show strong potency against BRD4, with IC_50_ values rangeing from 27 to 49 nM. In addition, they display a 30 − 120-fold BRD4 selectivity over BRD2, 3, and T, as well as a > 200-fold over non-BET family proteins. In a model of poly(I:C)-induced acute airway inflammation, compounds **63** and **64** could impede neutrophils accumulation without any apparent side effect [95]. In previous years, our laboratory also developed a selective BRD4 inhibitor, FL-411 (**65**), with IC_50_ values of 0.47 μM and 0.93 μM against BD1 and BD2 respectively [96]. We elucidated the relationship between the conformation and orientation of conserved residues (Gln85 and Pro82) and selectivity (Supplementary Fig. 1e). Compound **65** was shown to induce autophagy in tumor cells via the BRD4-AMPK-mTOR-ULK pathway and exhibits a striking anti-tumor activity in breast cancer xenograft models and zebrafish in vivo. Interestingly, a PET radiotracer [^11^C]MS417 (**67**), developed based on MS417 (**66**), similar to** [**^**18**^**F] 10**, can be used to assess the distribution and biological function of BET proteins in humans [97].

### BD-selective inhibitors

Although milestones have been achieved in the development of pan-BET inhibitors, and several drug candidates have progressed to clinical studies, their lagging, ambiguous mechanisms of action also raise concerns that perhaps clinical trials are running ahead of science [98]. Dawson et al. revealed that BD1 is implicated in steady-state gene expression, while both BD1 and BD2 are required in the rapid increase of gene expression stimulated by inflammation [99]. Further studies suggest that specific inhibition of BD1 can achieve similar anti-cancer efficacy as that of pan-BET inhibitors, whereas BD2 inhibitors show efficacy primarily in inflammatory and immune diseases. Even though the highly conserved sequence of BD1 and BD2 at the KAc recognition site makes it difficult to develop BD-selective inhibitors, the few non-conserved amino acid residues (e.g. Gln85, Ile146, Lys141 and Asp144 in BRD4-BD1 correspond to Lys378, Val439, Pro434 and His437 in BD2.) open the possibility of designing and exploiting selective inhibitors [38, 99, 100]. Accordingly, the development of new-generation inhibitors is focused on molecules that selectively bind to individual domains. Currently, several BD inhibitors, represented by **RVX-208** and **ABBV-744**, have been successfully developed and are currently under clinical trials [101, 102].

#### BD1-selective inhibitors

**Fig. 5:**
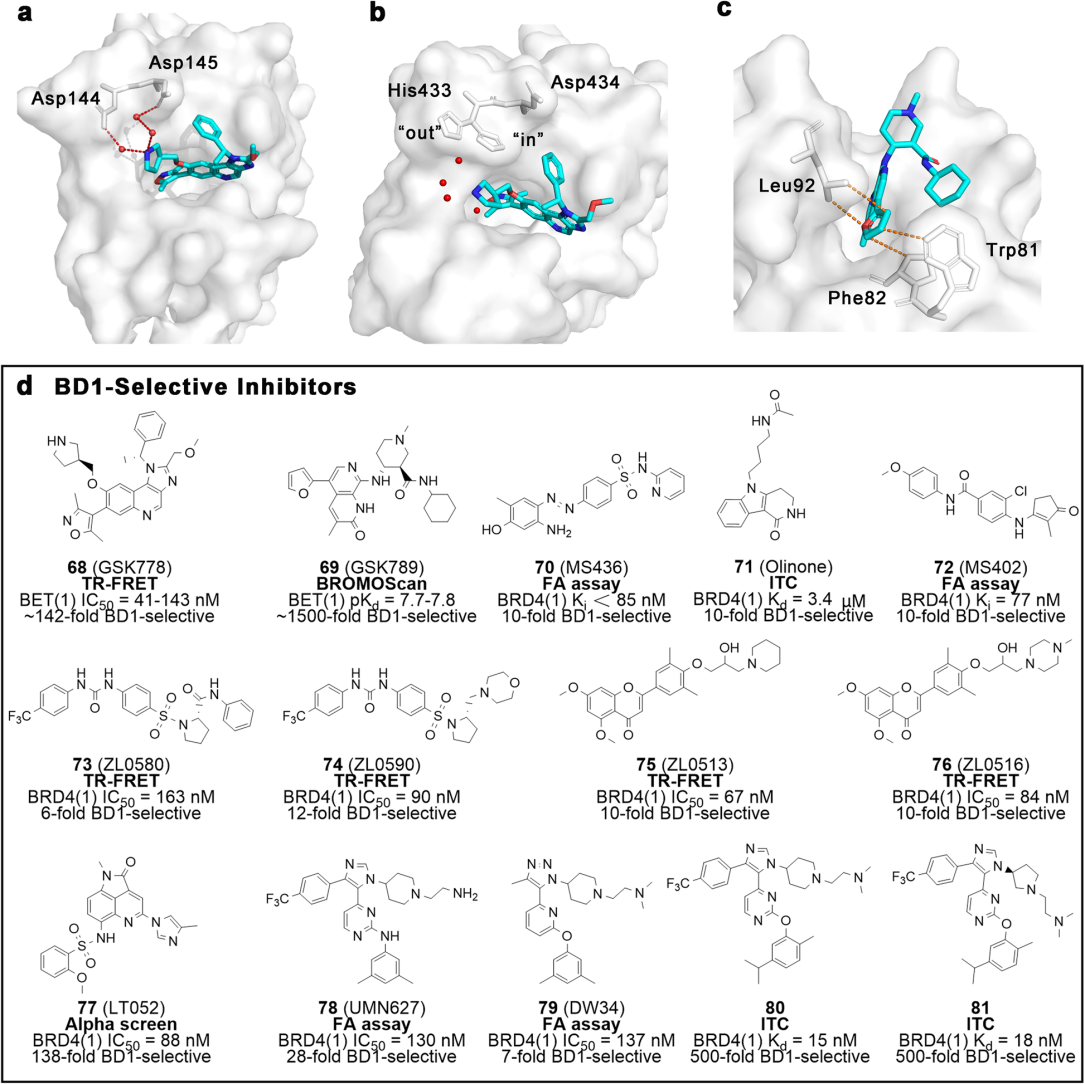
BD1-selective inhibitors. **a** Crystal structure of GSK778 bound to BRD4 BD1(PDB ID: 6SWN). **b** Crystal structure of GSK778 bound to BRD2 BD2 (PDB ID: 6SWO). **c** Crystal structure of GSK789 bound to BRD4 BD1 (PDB ID: 6Z7L). **d** Chemical structures of BD1-selective inhibitors **68** − **81**. Key residues are highlighted with grey sticks, and ligands are blue. Hydrogen bonds and hydrophobic interactions are shown in red and orange dashed lines, respectively. The activity test methods are bolded and the selectivity is labeled

GSK778 (**68**), yielded by introducing an additional pyrrolidine to compound **19** (Fig. 5), is a highly selective BD1 inhibitor (BRD4(1), IC_50_ = 41 nM) with a 143-fold selectivity over BD2. The nitrogen atom in pyrrolidine can form water-mediated hydrogen bonds with Asp144 (replaced with His433 in BRD2(2)) and Asp145, which may be responsible for its selectivity for BD1 (Fig. 5a-b). With the ability to efficiently displace chromatin-bound BRD4, compound **68** is considered to have the potential to be an alternative to pan-BET inhibitors in tumor therapy [99]. Also discovered by GSK, GSK789 (**69**, Fig. 5d) is 1000-fold more selective towards BD1 compared to BD2 [103]. Its X-ray structure suggests that furan is perfectly complementary to the hydrophobic cavity formed by Trp81, Phe82, and Leu92 in BRD4(1), while the C3′-amide of compound **69** conflicts spatially with His437 in BRD4(2) (Fig. 5c). Unsurprisingly, compound **69** possesses anti-proliferative and anti-inflammatory activities comparable to those of compound **4**.

Zhou’s team has focused on the development of BD1-selective inhibitors (Fig. 5d), and successively developed structurally diverse inhibitors including MS436 (**70**), Olinone (**71**) and MS402 (**72**) [104–106]. Compound **70** employs diazobenzene as its structural skeleton and can bind efficiently to BRD4(1) (K_i_ = 30 − 50 nM), with approximately tenfold selectivity for BD1 over BD2. High-resolution crystal structure data suggest that several critical water-mediated hydrogen bonds (e.g. between diazo and Pro82, amino and Asn140, sulfonamide and Lys91) are responsible for the selectivity toward BD1. In murine macrophages, compound **70** was shown to block nitric oxide production and IL-6 expression in a dose-dependent manner by interfering with the NF-κB pathway without apparent toxicity [104]. Meanwhile, **71** was generated by derivatization on the basis of MS7972. Compound **71** displays potent binding affinity to BRD4(1) with a K_d_ of 3.4 μM, whereas it shows only a weak affinity for BRD4(2) (K_d_ > 300 μM). The BRD4(1)-**71** complex demonstrates that triheterocyclic has a good reciprocal compatibility with Trp81, Pro82, and Ile146, while the acetyl chain has an optimal length to be inserted into the hydrophobic cavity and mimic the acetylated side chains of histones. However, in BRD4(2) the indole moiety collides with His437. Taken together, these may be the reasons contributing to the selectivity of compound **71** toward BD1 [105]. MS402 (**72**) was discovered during further studies on BD1-selective inhibitors and exhibits significant binding ability to BD1 (K_i_ = 77 nM), ninefold higher than that of BD2. In BD1, compound **72** is sandwiched between two hydrophobic regions, while the van der Waals force between cyclopentanone moiety and Ile146 and the hydrogen bond between the amide nitrogen and Gln85 contribute to the BD1 selectivity of this compound. Indeed, compound **72** can effectively block Th17 cell differentiation and offer a promising targeted therapeutic strategy to treat IBD [106].

Coincidentally, another Zhou’s team of researchers has also conducted continuous research on BD1-selective inhibitors and produced differently structured inhibitors (Fig. 5d). Compound ZL0580 (**73**) has a distinct structure and works as a selective BRD4(1) inhibitor with more than sixfold selectivity over BRD4(2) [107, 108]. Molecular docking demonstrates that **73** can achieve a more stable binding to BD1 compared to that of BD2. In a suppression model, **73** was shown to inhibit activation in a dose-dependent manner. Furthermore, this compound can efficiently suppress induced and basal HIV transcription in microglial cells. Mechanistic studies indicate that **73** can inhibit Tat (a protein critical for replication**)** transactivation and transcriptional elongation, and lead to chromatin remodeling with a repressor being induced at the HIV promoter. These efforts evidence the benefit of regulating BRD4 for HIV treatment in the field of epigenetics and provide a new lead compound for HIV epigenetic silencing. Based on **73**, further structural optimization was performed to achieve ZL0590 (**74**) which shows a tenfold selectivity to BRD4(1) over BRD4(2). Unlike conventional inhibitors that occupy the classical KAc recognition pocket, the allosteric binding of **74** is mainly located in the region enclosed by αB, αC, and THE BC loop [109]. The less conserved binding site and the great divergence in amino acid residues between BD1 and BD2 explain the selectivity of **74** toward BD1. Via oral administration, compound **74** was shown to suppress poly(I:C)-induced airway inflammation, attenuate inflammatory secretion and block acute airway inflammation. In parallel, other compounds, namely ZL0513 (**75**) and ZL0516 (**76**), also show high affinity for BRD4(1) with an IC_50_ of 67 nM and 84 nM respectively [110]. Both compounds are approximately tenfold more selective toward BD1 over BD2, mainly due to the formation of water-mediated hydrogen bonds between the side chain hydroxyl group and the Asn93 residue (which is replaced by glycine in BD2). Moreover, these two compounds significantly improve airway inflammation of mice induced by poly(I:C) and show outstanding drug metabolism and pharmacokinetics (DMPK) properties.

Chen et al. reported LT052 (**77**), a recognized highly selective BD1 inhibitor (Fig. 5d). This molecule effectively alleviates acute gouty arthritis by regulating the BRD4/NF-κB/NLRP3 signaling pathway [111]. IC_50_ values from Alphascreen assay demonstrate that compound **77** is 100-fold more selective toward BD1 over BD2, thanks to the spatial mutual repulsion between the rigid methylimidazole of **77** and the H437 residue of BD2. In recent years, a series of BD1-selective inhibitors (**78** – **81**) with similar scaffolds have been reported by Pomerantz’s group [112–114]. All of them show a high preference for BD1 with selectivity ranging from tens to hundreds of times over BD2. Water-bridged hydrogen bonds and displacement of structured waters in the pocket contribute to the BD1 selectivity of these compounds. These results provide novel chemotype leads for the study of selective BD1 inhibitors for the treatment of tumors and inflammation, as well as function as a reference for follow-up structural design.

#### BD2-selective inhibitors

**Fig. 6:**
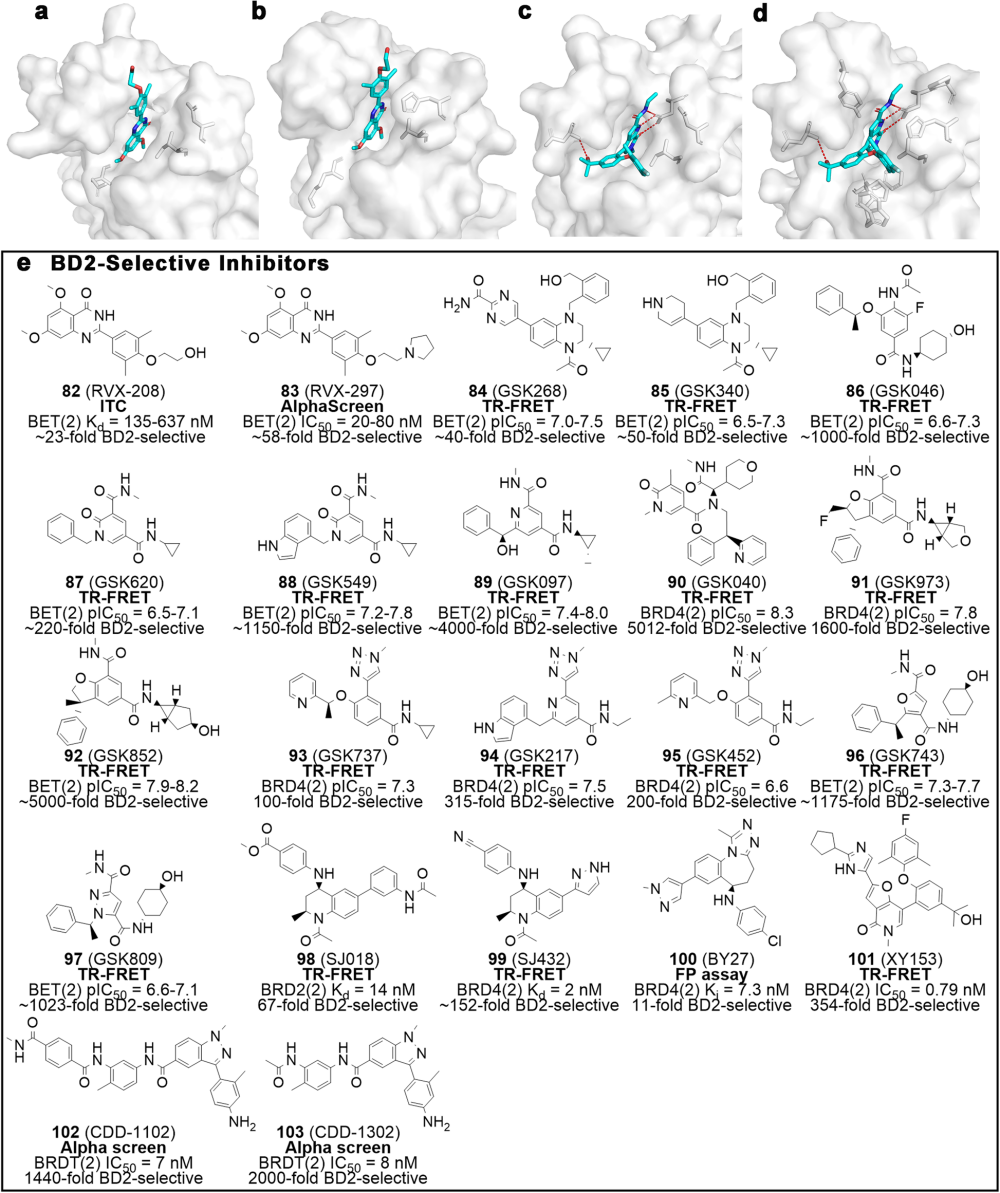
BD2-selective inhibitors. **a** Crystal structures of RVX-208 bound to BRD4 BD1(PDB ID: 4MR4). **b** Crystal structures of RVX-208 bound to BRD2 BD2 (PDB ID: 4MR6). **c** Crystal structures of ABBV-744 bound to BRD2 BD1 (PDB ID: 6ONY). **d** Crystal structures of ABBV-744 bound to BRD2 BD2 (PDB ID: 6E6J). **e** Chemical structures of BD2-selective inhibitors **82** − **103**. Key residues are highlighted with gray sticks, and ligands are blue. Hydrogen bonds are shown in red dashed lines. The activity test methods are bolded and the selectivity is labeled

RVX-208 (RVX000222 or apabetalone, **82**, Fig. 6), the most studied selective inhibitor of BD2, was originally developed by Resverlogix Corporation for the treatment of atherosclerosis-related diseases and is effective in increasing ApoA1 concentration. Subsequently, compound **82** was identified by the Filippakopoulos’ team as a binding partner of BD2 with more than 20-fold selectivity over BD1 [19, 115]. The characteristic His433 residue in BRD2(2) flips into the KAc recognition pocket opposite the benzene ring, resulting in a tightening of the pocket that allows the inhibitor to fit more closely to the pocket, which may underlie its selectivity (Fig. 6a-b). Recently, results from a phase III clinical trial (BETonMACE study) of RVX-208 in subjects with high-risk type 2 diabetes mellitus with coronary artery disease showed that **82** reduces risk of major adverse cardiovascular events (MACE) and hospitalization due to heart failure [116]. RVX-297 (**83**), a molecule structurally similar to **82**, is twice as selective for BD2 as **82**, with more than 50-fold selectivity towards BD2 over BD1 [117]. Importantly, compound **83** shows considerable therapeutic efficacy in preclinical models of acute inflammation and autoimmunity [118]. Another BD2-selective inhibitor advancing to clinical trials is **49**, which is ultra-selective for BD2 [85, 86]. Crystal structures suggest that the amide of **49** stretches into the channel formed by His433, Tyr386 and Pro430 in BD2, which is not allowed in BD1. Furthermore, the Val435 of BD2 can accommodate the 2,6-dimethylphenyl group without affecting binding, whereas the larger Ile162 in BD1 does not allow this to occur (Fig. 6c-d). For the treatment of AML, the efficacy of **49** is comparable to that of pan-BET inhibitors, and its therapeutic index remains to be improved [119]. A phase I clinical trial of **49** in subjects with relapsed or refractory AML was discontinued due to strategic reasons. Surprisingly, both **82** and **49** exhibit COVID-19 therapeutic potential by reducing *ACE2* expression, suppressing SARS-CoV-2 replication, and/or binding stably to the M^pro^ [120–122].

GSK has continuously reported a number of BD2-selective inhibitors (Fig. 6e), including tetrahydroquinoxaline [123], pyridinone [124], picolinamide [125], and dihydrobenzofuran [126, 127]. Amidopyrimidine GSK268 (**84**) and tetrahydropyridine GSK340 (**85**) are potent pan-BET inhibitors with IC_50_ values ranging from 32 to 100 nM [123]. Both are BD2-selective inhibitors with an approximately 50-fold selectivity to BD2 over BD1. X-ray crystal structures indicate that **84** and **85** share a similar binding pattern to BRD2(2), in which the 6-substituent extends into the ZA channel to make a π interaction with Trp370, in addition to which **85** can form an extra hydrogen bond with His433. Both inhibitors are capable of permeabilizing cells and effectively inhibiting the release of monocyte chemattractant protein 1 (MCP-1) induced by LPS. Through HTS and exhaustive optimization, GSK046 (iBET-BD2, **86**) has drawn the attention of researchers. This compound exhibits extremely high selectivity to BD2 with a 1000-fold selectivity over BD1. Its excellent selectivity and activity are owing to the extensive hydrophobic interaction of its 3-position benzyl substituent and 5-position cyclohexanol ring with His433 and Pro430 in BRD2-BD2 [99, 128]. GSK620 (**87**) and GSK549 (**88**) were constructed based on **86** via a scaffold hopping approach [124], and exhibit 200-fold and 1000-fold selectivity to BD2 over BD1, respectively. With satisfactory PK properties, the two inhibitors reduce MCP-1 response in a dose-dependent manner, as well as serve as useful tools against inflammatory and autoimmune diseases. To improve solubility, a series of molecules containing a picolinamide template were designed. Among them, GSK097 (**89**) achieves a perfect balance of activity, selectivity, and solubility [125]. This compound displays excellent inhibitory activity to BD2 with an IC_50_ of approximately 12 nM, as well as a 4000-fold selectivity over BD1 and > 1000 μg/mL solubility in fasted state simulated intestinal fluid (FaSSIF). As of today, GSK040 (**90**) is the most selective BD2 inhibitor, with an astounding 5000-fold selectivity for BD2 over BD1 [129]. The Co-crystal structure suggests that **90** and BD2 are perfectly complementary in space, while the multiple interactions guarantee its high selectivity. Briefly, the reasons contributing to its ultra-high selectivity include the stacking of methyl amide with Leu381, Lys378-related interactions and water structure changes, bulk substituents of the α-carbon of glycine, and sandwiching His433 with the bis-aryl moiety. Dihydrobenzofurans GSK973 (**91**) [126]and GSK852 (**92**) [127]are both BD2 selective inhibitors, with more than 1000-fold selectivity over BD1. Both inhibitors exhibit not only exceptional MCP-1 inhibitory activity, but also good solubility and PK profiles. GSK737 (**93**), GSK217 (**94**), GSK452 (**95**) are analogs of the above compounds (e.g. **86** and **88**) obtained by replacing the acetamide moiety of the structure with a 1,2,3-triazole template [130]. This optimization strategy is designed to reduce the genotoxicity of inhibitors. Notably, all three compounds have favorable selectivity, potencies and pharmacokinetic profiles in rats. Among the later generation of GSK BD2 inhibitors, the furan GSK743 (**96**) and pyrazole GSK809 (**97**) exhibit outstanding solubility and over 1000-fold selectivity for BD2 [131]. With favorable physicochemical properties, excellent selectivity, synthetic accessibility, and no obvious off-targets, the two compounds represent a new class of BD2 inhibitors that are more compatible with clinical needs.

Potter’s team successively developed the BD2-selective inhibitors SJ018 (**98**) and SJ432 (**99**), employing THQ as their scaffold [132] (Fig. 6e). Compound **99** was shown to effectively down-regulate MYC protein levels in neuroblastoma cell lines without rebound more effectively than compound **1**. In pediatric neuroblastoma xenograft models, **99** can slow tumor growth accompanied by reduced c-MYC, loss of BRD4 and increased HEXIM1, with no significant toxicity. Compound **99** demonstrates a 152-fold selectivity for BD2 versus BD1. Such selectivity is associated with benzonitrile moiety, which can form π-π stacking with His433 and bind tightly to the pocket. These outcomes reaffirm that the development of highly BD-selective inhibitors is an efficacious strategy to achieve high-efficiency, low-toxicity anti-tumor candidates.

Lead compounds BY27 (**100**) [133]and XY153 (**101**) [134] show a preference for BD2, both of which form water-mediated hydrogen bonds with His433. These two compounds exhibit potent anti-proliferative activity against MV4-11, providing a valuable reference for the development of candidates against AML. Impressively, Matzuk et al. developed a series of BRDT(2) inhibitors via a DNA-encoded chemistry technology (DEC-Tec) platform [134]. Among these compounds, CDD-1102 (**102**) and its truncated analog CDD-1302 (**103**) can potently inhibit BRDT(2) at nanomolar levels with well over 1000-fold selectivity over BRDT(1), creating a practical option for the development of non-hormonal male contraceptives.

In summary, specific interactions between Asn140 and Asp144 in BD1 and inhibitors lead to enhanced selectivity, while the selectivity of compounds for BD2 is dependent on interactions with His433. Selective inhibition of BDs enables us to better understand the function and efficacy of each domain, as well as to circumvent unintended toxicities, which is bound to be the focus of BET inhibitor development in the years to come.

### Bivalent inhibitors

**Fig. 7:**
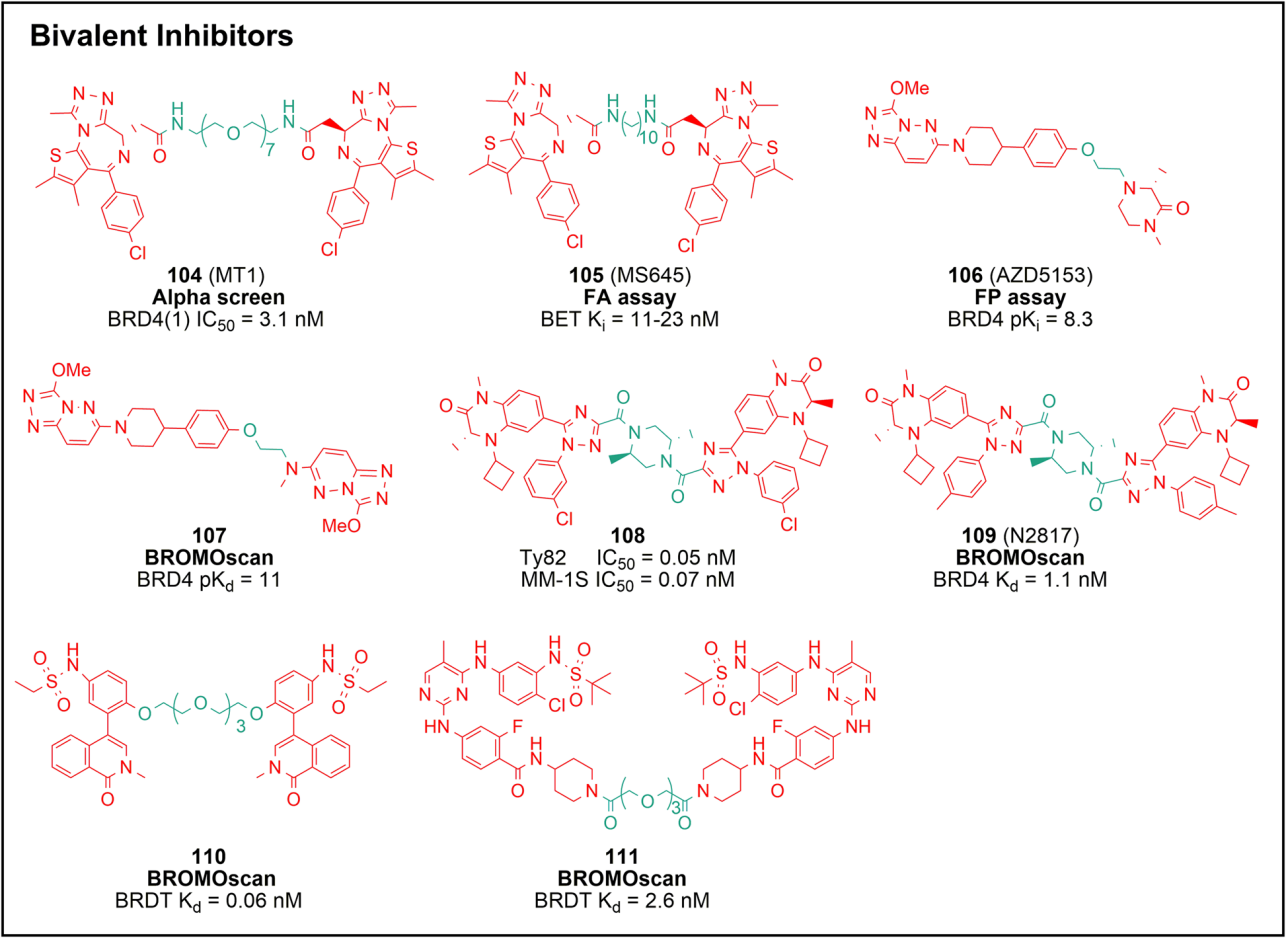
Chemical structures of bivalent inhibitors **104** − **111**. The activity test methods are bolded. The pharmacophore part is shown in red, and the linker is shown in green

In recognition of the fact that BET contains two separate BDs, studies have focused on the development of bivalent inhibitors to improve potency. MT1 (**104**) is the first bivalent BET inhibitor, consisting of a homodimer of compound **1** and a polyethylene glycol (PEG) spacer, with full structural symmetry [135] (Fig. 7). Mechanistically, compound **104** binds to two tandem BDs simultaneously in an intramolecular manner and each triazole moiety occupies the KAc pocket and forms a hydrogen bond with the conserved asparagine (Supplementary Fig. 1j). The inhibitory activity of **104** against BRD4(1) (IC_50_ = 3.09 nM) is significantly greater than that of **1** (IC_50_ = 20.9 nM), while its cellular activity is more than 100-fold stronger compared with that of compound **1**. In vivo, compound **104** can dramatically reduce the leukemic burden and improve overall survival at a dose of 22.1 μmol/kg compared to **1** (44.2 μmol/kg). However, **104** administered at a dose of 44.2 μmol/kg can induce weight loss in mice, which is indicative of drug toxicity.

Zhou’s group prepared a series of bivalent BET inhibitors by linking two molecules of **1** homolog with spacers (e.g. alkyl, PEG and benzene) of different lengths and rigidities [136] (Fig. 7). Among these inhibitors, MS645 (**105**), employing a 10-carbon alkyl as the linker, was shown to inhibit TNBC cell proliferation by effectively interfering with BRD4 binding to the transcription enhancer or mediator. This work elucidates the important relationship between linker length, composition, and rigidity of bivalent inhibitors on their pharmacological effectiveness and suggests a strategy for maximizing inhibition of BRD4 activity for the management of rapidly growing solid tumors.

The asymmetrical compound **AZD5153** (**106**) and the pseudosymmetrical compound **107** were developed by AstraZeneca via an androgen receptor downregulation program (Fig. 7). Both can simultaneously bind to BD1 and BD2 in a single BET protein via an *in cis* binding mode. In mice bearing MV4-11 xenografts, compound **106** can reduce the expression of c-MYC even at free plasma concentrations lower than 0.2 μM and lead to an almost complete tumor regression at a dose of 10 mg/kg. Compound **107** exhibits powerful cell-killing effects in MV4-11 and MM-1S cells (pIC_50_ = 9.5), with an efficacy of three orders of magnitude higher in comparison to that of compound **4** [137–139].

To optimize the undesirable PK properties due to the long linker, bivalent inhibitors harboring hydrophilic 2,5-dimethylpiperazine as the spacer were designed (Fig. 7). Orally available inhibitors **108** and N2817 (**109**) display excellent activity in vitro and in vivo [140, 141]. Compound **108** potently inhibits MV4-11 with an average IC_50_ value of 0.77 nM, while it reduces tumor growth at an oral dose of 0.6 mg/kg, outperforming compound **48** at the same dose. Interestingly, inhibition of the incidental transcription initiation factor TFIID subunit 1 (TAF1) by **109** contributes to its powerful anti-proliferative capacity, with IC_50_ values ranging from 0.19 to 228 nM in a variety of solid tumor cell lines. Symmetrical compounds **110** and **111**, developed from non-selective isoquinolinone and diaminopyrimidine monovalent inhibitors, show superior selectivity and activity against BRDT over univalent inhibitors. Overall, these findings suggest that the distinct plasticity of the BET bromodomain after inhibitor-induced dimerization facilitates the selectivity of bivalent inhibitors within the BET family [142].

Collectively, these works highlight an intriguing design concept, namely the simultaneous targeting of two individual structural domains with a bifunctional small molecule, providing a promising prototype for the development of ligands for other multi-domain proteins. Although bivalent inhibitors exhibit superior BET binding and pharmacological effects than monovalent ones, the resulting deterioration in their physicochemical properties, metabolic instability, and increased toxicity should also be considered.

### Dual-target inhibitors

Combinations have emerged as a proven approach to maximize efficacy, with dual-target inhibitors gradually attracting significant interest among medicinal chemists [38, 143]. Concurrent suppression of two individual targets often leads to synergistic effects and counteracts drug resistance. Additionally, the administration of dual-target inhibitors can also reduce the dosage and improve patient compliance compared to the combined use of two separate drugs [143]. The co-inhibiting of BET and other targets has been considered a promising strategy to treat various types of cancers. These other targets include, but are not limited to histone deacetylases (HDACs), polo-like Kinase 1 (PLK1), phosphatidylinositol 3-kinase (PI3K), poly(ADP-ribose) polymerase 1 (PARP1), and CDKs.

**Fig. 8:**
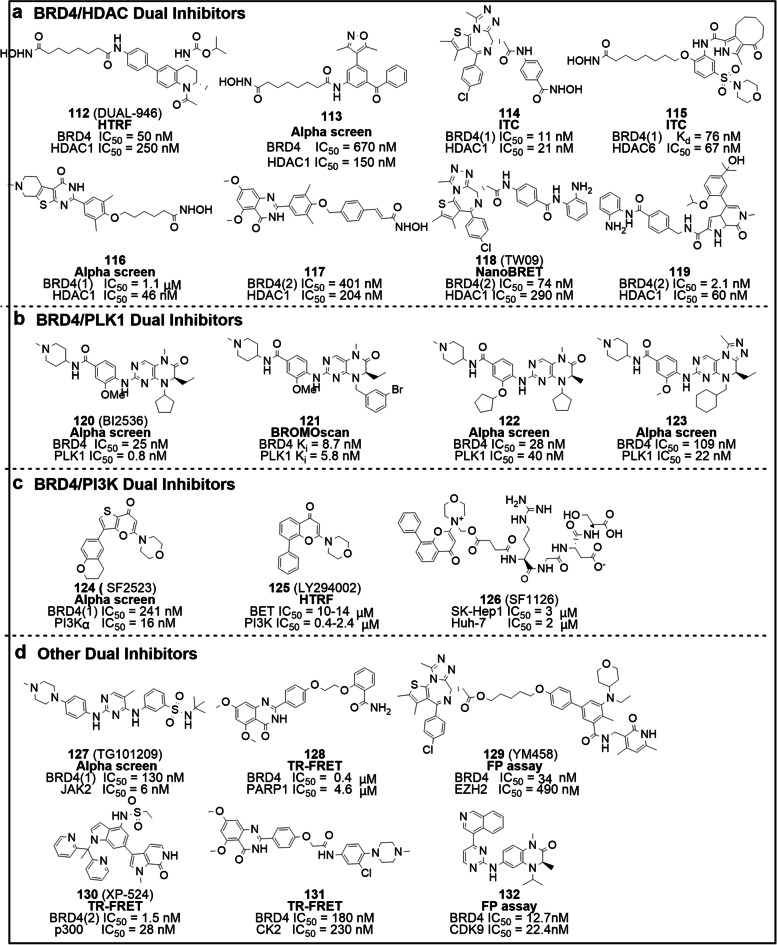
Dual inhibitors based on BRD4. **a** Chemical structures of BRD4/HDAC dual inhibitors **112** − **119**. **b** Chemical structures of BRD4/PLK1 dual inhibitors **120** − **123**. **c** Chemical structures of BRD4/PI3K dual inhibitors **124** − **126**. **d** Chemical structures of other dual inhibitors based on BRD4 **127** − **132**. The activity test methods are bolded

Several reports have validated the concept of combined inhibition of BRD4 and HDAC as a synergistic therapy against tumors [144]. For instance, isohydroxamic acid derivatives of I-BET726 (DUAL946, **112**), I-BET151 (**113**), ( +)-JQ1(**114**), XD14(**115**) (Fig. 8a). Compound **112**, as expected, effectively inhibits BRD4 (BD1 IC_50_ = 50 nM) and HDACs (IC_50_ = 0.25 − 35 μM) [145]. By down-regulating c-MYC expression and augmenting acetylation of histone H4, compound **112** was found to suppress the growth of NMC and AML cell lines, but regrettably no synergistic effect was observed compared to the parent compound. Meanwhile compound **113** exhibits submicromolar inhibitory activity against BRD4 and HDAC1, and slightly inferior anti-proliferative activity in K562 and MV4-11 in vitro when compared to that of compound **1**. Docking models of **113** bound to BRD4(1) reveal that the 3,5-dimethylisoxazole is packed in the pocket by mimicking KAc, forming hydrogen and water-mediated hydrogen bonds with Asn140 and Tyr97, respectively. The benzoyl group of **113** enters the “WPF shelf” and the alkyl linker extends to the ZA channel. Besides, in HDAC1 the compound is immobilized at the binding site by stable chelation of hydroxamic acid with a zinc atom, forming hydrogen bonds with Tyr297, His131 and His132, while the cap fragment interacts with the hydrophobic region of the protein surface [146]. Compound **114** is a robust pan-BET/HDAC dual target inhibitor with IC_50_ values ranging from 11 to 316 nM for BET and 21 to 192 nM for HDAC [147]. In terms of BET and HDAC inhibition, **114** achieves a favorable balance and shows acceptable metabolic stability. In a Capan-1 xenograft model, intraperitoneal injection of **114** was shown to effectively reduce tumor growth in a dose-dependent manner, with a TGI value of 87.7% at a dose of 20 mg/kg. Indeed, the activity of compound **114** was superior to that of **1** and SAHA administered alone or in combination. With low nanomolar activity against BET and moderate submicromolar activity against HDAC, compound **115** is the first XD14-based dual HDAC/BET inhibitor [148]. This compound demonstrates broad-spectrum anti-tumor activity in six different leukemia cell lines. Notably **115** is well-tolerated in a zebrafish model at concentrations lower than 50 μM. Our laboratory has developed a BRD4/HDAC dual inhibitor candidate, compound **116** through a four-step strategy [149]. Compound **116** can effectively inhibit BRD4 and HDAC and induce autophagic death in colorectal cancer cells. Inspiringly, **116** can address the resistance issue of specific HDAC inhibitors by inhibiting the IL6-JAK-STAT signaling pathway. Selective HDAC/BRD4 dual inhibitor **117** binds more preferentially to BD2 relative to BD1(over 100-fold), and its anti-proliferative capacity against AML cell lines is superior to that of RVX-208 and SAHA [150]. Finally, the benzamide dual-target inhibitor TW09 (**118**) serves as a scaffold for the selective targeting of HDAC1, while compound **119** exerts anti-tumor synergistic effects [151–153].

Simultaneous inhibition of PLK1 and BRD4 is also an effective therapeutic strategy for diseases driven by these proteins. BI2536 (**120**) and fine-tuned derivatives **121** − **123** demonstrate powerful inhibitory activity against PLK1 and BRD4 and anti-proliferative activity against hematological malignant cells [154–157] (Fig. 8b). Several Phase I and Phase II clinical trials of **120** for the treatment of patients with advanced solid tumors or hematological malignancies have been completed, confirming its efficacy and safety [154]. *MYC* gene expression is tightly regulated by PI3K and BRD4, therefore concomitant inhibition of PI3K and BRD4 blocks *MYC* expression and activation, leading to decreased tumor growth and metastasis. SF2523 (**124**) was initially reported as a potent pan-PI3K inhibitor, showing a high affinity towards BRD4 (Fig. 8c). Compound **124** administered alone was shown to significantly inhibit tumor growth in the Panc02 carcinoma model with equivalent efficacy and less toxicity compared to the combination of **1** and BKM120 [158–160]. Quercetin derivative LY294002 (**125**) exhibits moderate inhibitory activity against PI3K and BET [161], while SF1126 (**126**) is a prodrug prepared by coupling **125** with an RGDS peptide. Compound **126** alone or in combination with sorafenib can inhibit hepatocellular carcinoma (HCC) proliferation, induce apoptosis and cell cycle arrest, displace BRD4 from the *MYC* transcriptional start point, and inhibit HCC growth in vivo by interfering with the PI3K/AKT/mTOR, and Ras/Raf/MAPK singling pathways. A phase I study suggested **126** is well tolerated in patients suffering from advanced solid tumors and B-cell malignancies [162, 163].

JAK2 inhibitor TG101209 (**127**) also exhibits potent BRD4 inhibitory activity with an IC_50_ of 130 nM, which is attributable to the aminopyrimidine moiety that can form hydrogen bonds with Asn140 and Pro82, respectively [143]. Other dual inhibitors (Fig. 8d), such as the BRD4/PARP1 inhibitor ADTL-BPI1901 (**128**) [164], the BRD4/EZH2 inhibitors YM458 (**129**) [165], the BRD4/p300 inhibitor XP524 (**130**) [166], the BRD4/CK2 inhibitor **131** [167], and the BRD4/CDK9 inhibitor **132** also achieve synergistic benefits and overcome tumor resistance by simultaneously inhibiting two separate pathways implicated in the cancer [168].

### PROTACs

**Fig. 9:**
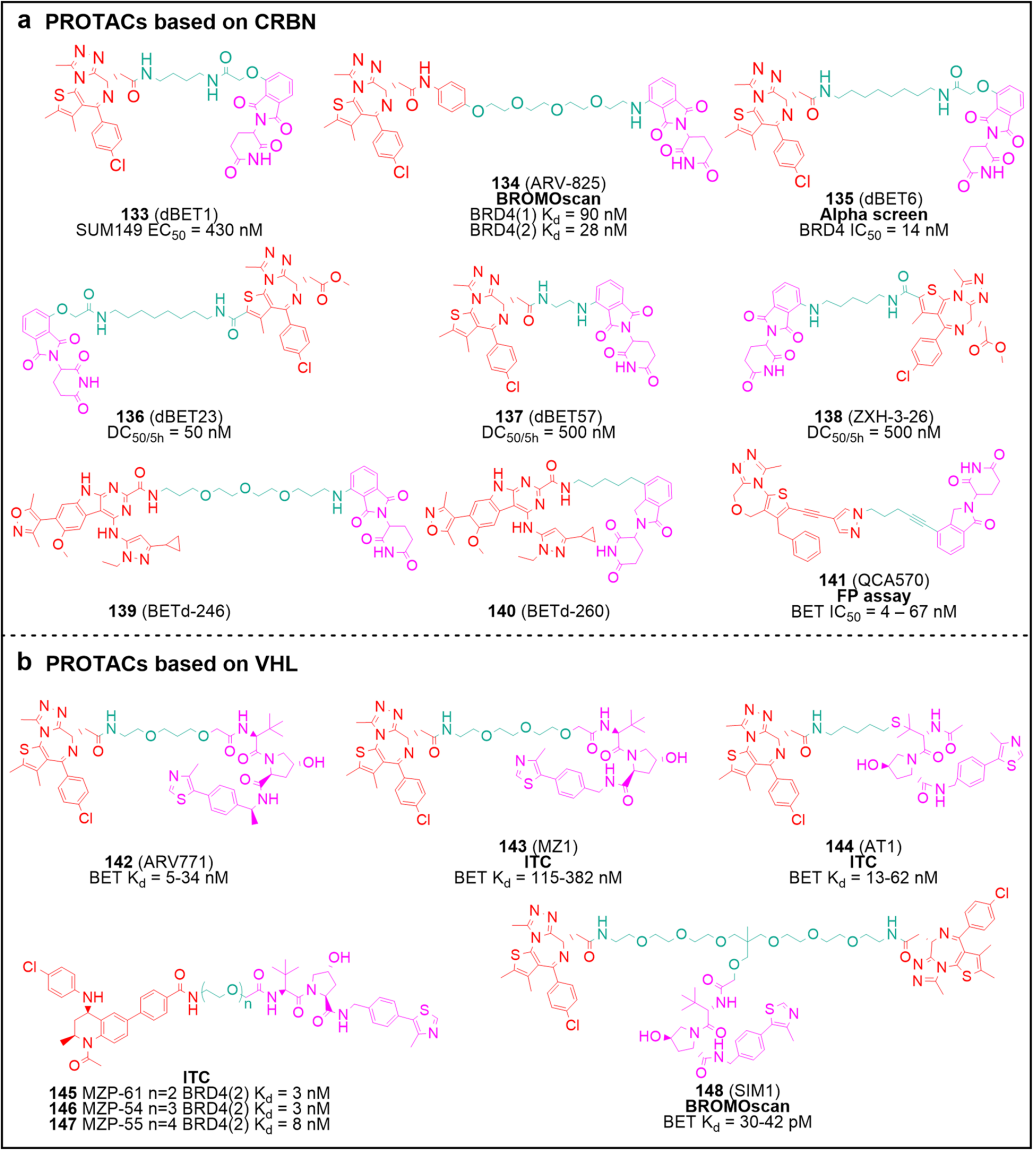
PROTACs. **a** Chemical structures of CRBN-based PROTACs **133** − **141**. **b** Chemical structures of VHL-based PROTACs **142** − **148**. The warheads are shown in red, the anchors in purple, and the spacers in green. The activity test methods are bolded

Recently, proteolysis targeting chimeras (PROTACs) were described as an effective approach to induce protein degradation. PROTACs are typically designed as heterobifunctional molecules comprising a warhead that binds the protein of interest (POI), an anchor for the E3 ubiquitin ligase, and a spacer that tandemly links them [169, 170]. Owing to the growing clarity of the biological function of BET proteins and the influx of inhibitors into clinical studies, PROTACs have also been applied to target BET proteins. dBET1 (**133**) was the first reported BET-targeting PROTAC, designed as compound **1** conjugated with thalidomide which can be anchored to cereblon (CRBN) [171] (Fig. 9a). When MV4-11 cells were treated with 100 nM of compound **133** for 18 h, endogenous BRD4 decreased by 85%. Similarly, compound **133** treatment for 18 h resulted in BRD4 depletion in SUM149 cells with an EC_50_ value of 430 nM. Compared with **1**, compound **133** induces more apoptosis and exerts a more pronounced anti-proliferative activity in MV4-11 cells, highlighting that degrading the entire BET protein is perhaps more promising than inhibiting individual BDs, facilitating the development of PROTACs. Another CRBN-based PROTAC, ARV-825 (**134**) (Fig. 9a), was derived from compound** 5** and employed PEG as the linker, enabling the complete degradation of BRD4 in Burkitt’s lymphoma (BL) cells with a 50% of maximum degradation (DC_50_) < 1 nM. Compound **134** inhibits BRD4 activity and down-regulates c-MYC protein level, displaying superior apoptosis induction and proliferation suppression than small molecule inhibitors [172]. dBET6 (**135**) is a highly cell-permeable PROTAC with a slightly higher BRD4 binding affinity (IC_50_ = 14 nM) than **133**, and was shown to significantly improve the survival of mice bearing T cell acute lymphoblastic leukemia (T-ALL) at the dose of 7.5 mg/kg [173]. Compounds dBET23 (**136**), dBET57 (**137**), and ZXH-3–26 (**138**) demonstrated that the binding of ligases to substrates is plastic and can adjust their conformation according to the length and the attachment site of the linker, while the plasticity of inter-protein contacts provides a basis for the development of highly selective PROTACs [174]. Wang’s laboratory reported two distinct types of CRBN-based PROTACs BETd-246 (**139**) [175], BETd-260 (**140**) [72] and QCA570 (**141**) [176] (Fig. 9a)**,** which built on their previously described BET inhibitors compound **31** and QCA276. After treating TNBC cell lines with **139** at 30 − 100 nM for 1 h, it was shown that BRD2, 3 and 4 were abrogated. In parallel, compound **140,** a further optimized degrader, was shown to dramatically reduce BRD4 level at 30 pM and to inhibit RS4-11 with an IC_50_ value of 51 pM. Admirably, after much effort, researchers discovered the extremely powerful degrader **141**, which could effectively degrade BRD3 and BRD4 in MV4-11 and RS4-11 cell lines at concentrations as low as 10 nM. In addition, **141** exhibits picomolar-level inhibitory vigor against MV4-11 with an IC_50_ of 8.3 pM, making it the most powerful BET-targeting PROTAC by far. These compounds underline the capacity of PROTACs to inhibit target proteins to a greater extent than their parent compounds, at least as evidenced by in vitro assays.

Compounds **142 − 148** are von Hippel-Landau (VHL) E3 ligase-based degraders (Fig. 9b). ARV771 (**142**) treatment of 22Rv1 cells causes remarkable degradation of BRD2, 3 and 4 (DC_50_ < 5 nM), accompanied by depletion of c-MYC (IC_50_ < 1 nM). The activity of this compound is greater than 10 and 100-fold higher than that of compounds **1** and **5**, respectively. In addition to a more pronounced apoptotic effect than their parental inhibitors and a comparable anti-tumor effect in vivo, compound **142** also down-regulates full-length AR. This finding provides valuable advances for the development of CRPC therapeutics [177].

In Ciulli’s works, MZ1 (**143**) was prepared by tethering compound** 1** (for BD recruitment) and VHL-1(a VHL ligand) together with a PEG linker [178] (Fig. 9b). Compound **143** is a relatively BRD4-selective degrader and was shown to deplete more than 90% of this BET protein in HeLa cells at concentrations as low as 1 μM. Following this, researchers illustrated the crystal structure of the MZ1-BRD4(2)-VHL ternary complex and redesigned a highly selective BRD4 degrader AT1 (**144**) that could effectively degrade BRD4 at 1 − 3 μM relative to the ignorable activity against BRD2 and BRD3 [179]. To clarify the relationship between BET inhibitor activity and degrader efficacy, they developed several BET degraders, namely MZP-61 (**145**), MZP-54 (**146**) and MZP-55 (**147**) with **37** as the parental compound [180] (Fig. 9b). Their data showed that these **37-**based degraders did not display higher degradation performance and, on the contrary, were less effective than the JQ1-based degraders. These results suggest that a more potent inhibitor is not a prerequisite to generate a superior PROTAC, underscoring the significance of the formation of a stable ternary complex. Inspired by the crystal structure of the MZ1-BRD4(2)-VHL ternary complex and aiming to enhance targeted degradation, they recently developed the trivalent PROTAC SIM1 (**148**) on the foundation of **143** [181]. Compound **148** exhibits enhanced and longer-lasting BET degradation benefits compared to those of **143**, with DC_50_ values ranging from 0.7 to 3.3 nM, a more significant BET down-regulation and c-MYC repression in BET-sensitive 22Rv1 cells. As with the bivalent BET inhibitors described above, **148** binds to BD1 and BD2 in a *cis* manner, forming a complex with VHL and BET (BD1 and BD2) in a 1:1:1 ratio. Unexpectedly, despite such large bulk, compound **148** shows remarkable bioavailability and stability in mice.

Differing from occupancy-driven inhibitors, PROTACs provide multiple advantages such as high efficiency, low toxicity, small dose delivery, and improved drug resistance. However, the accompanying low permeability and bioavailability also pose new challenges. Currently, only two E3 ubiquitin ligases (CRBN and VHL) have been implicated in the proof-of-concept of BET-targeting PROTACs. Designing PROTACs tailored to the tumor-specific, highly expressed POIs and E3 ligases to minimize off-target effects is a longstanding challenge and the foundation for providing tangible therapeutic benefits.

## Non-BET inhibitors

Motivated by the growing sophistication of BET functional studies and inhibitors development, several scholars have manifested an ever-increasing enthusiasm to characterize non-BET inhibitors. Scores of non-BET BD ligands have been designed as chemical probes or inhibitors to elucidate unclear biological functions and to facilitate drug discovery [182, 183].

### CBP/p300 inhibitors

**Fig. 10:**
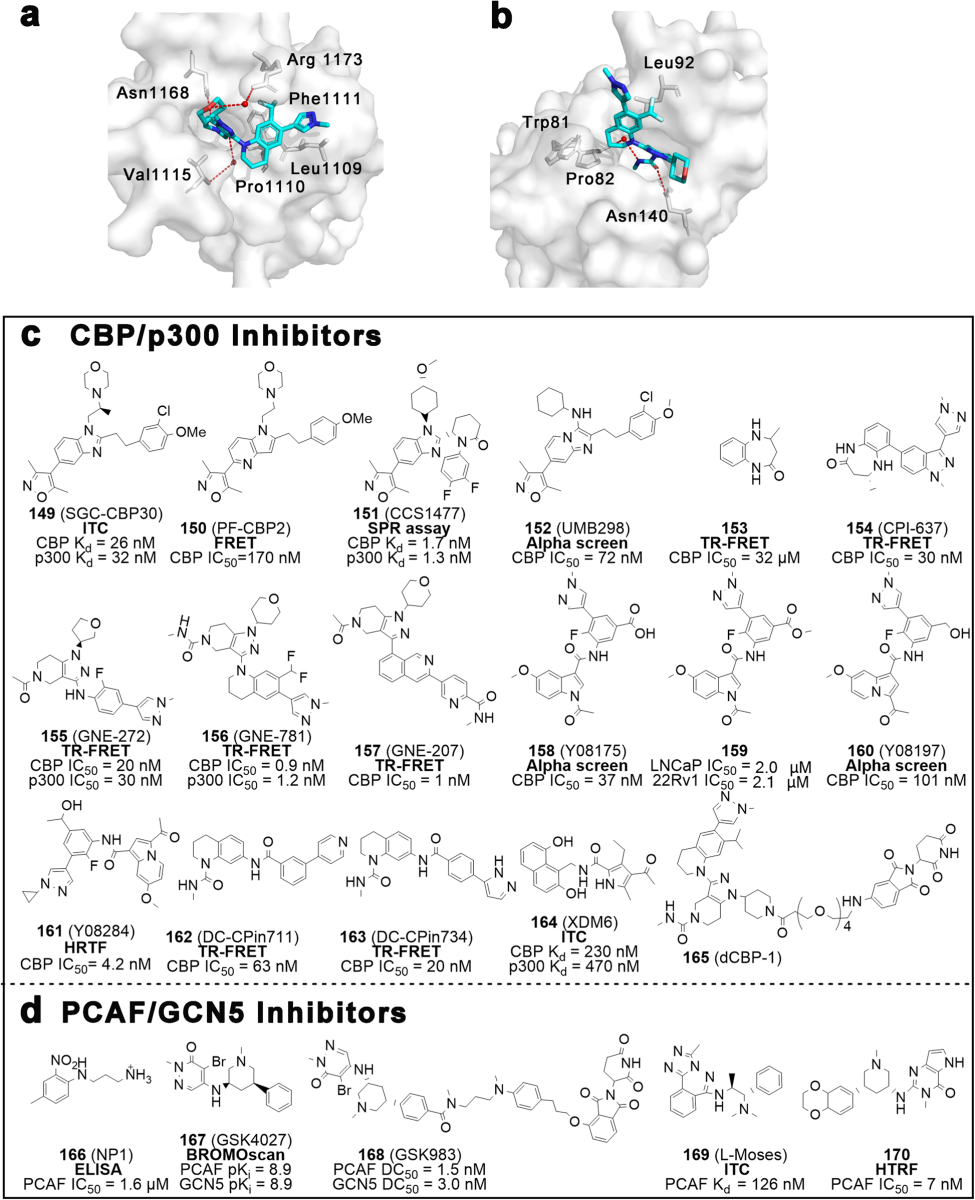
CBP/p300 and PCAF/GCN5 inhibitors. **a** Crystal structures of GEN-781 bound to CBP (PDB ID: 5W0E). **b** Crystal structures of GEN-781 bound to BRD4 BD1 (PDB ID: 5VZS). **c** Chemical structures of CBP/p300 inhibitors **149** − **165**. **d** Chemical structures of PCAF/GCN5 inhibitors **166** − **170**. Key residues are highlighted with gray sticks, and ligands are blue. Hydrogen bonds are shown in red dashed lines. The activity test methods are bolded

Outside of the BET family, CBP/p300 are the most well-studied BCPs, with a high degree of biological homology. In recent years, small molecule inhibitors of CBP/p300, particularly represented by dimethylisoxazoles, were dramatically developed (Fig. 10c). Structural Genomics Consortium (SGC) characterized the compound SGC-CBP30 (**149**) in 2014 after a sequence of structural optimization, which exhibits potent affinity and selectivity (CBP K_d_ = 26 nM, p300 K_d_ = 32 nM), being 40-fold and 250-fold more selective for CBP over BRD4(1) and BRD4(2) respectively. Compound **149** was shown to effectively inhibit doxorubicin-induced p53 activity and IL-17A secretion by Th17 cells. In comparison to pan-BET inhibitors, **149** potentially has fewer side effects and a better safety profile [184, 185]. Conversion of benzimidazole to pyridopyrrole (PF-CBP2, **150**) eliminates the water-mediated hydrogen bond with BRD4 Pro82 and improves the selectivity and affinity for CBP [186]. Based on its selectivity (100-fold over BRD4) and effectiveness (CBP IC_50_ = 0.17 μM), compound **150** may be employed as a lead compound for further drug development. CCS1477 (**151**), found as a highly efficient, selective and orally available inhibitor, is the only CBP inhibitor to advance to clinical studies to date [187]. Two Phase I/IIa clinical trials aiming to evaluate the safety, tolerability, PK and biological activity of **151** in patients with metastatic CRPC, advanced solid tumors (NCT03568656) and hematological malignancies (NCT04068597) are scheduled to be completed in March 2024.

Zhang’s team has efficiently synthesized a series of CBP/p300 inhibitors with imidazo[1,2-a] pyridine as the scaffold in a two-step approach (Fig. 10c), providing synthetic convenience for rapid and comprehensive SAR studies. Among them, UMB298 (**152**) was shown to exhibit comparable biological activity and selectivity to the reported inhibitors, with a 72-fold selectivity toward CBP over BRD4 [188]. Constellation Pharmaceuticals, in collaboration with Genentech Inc, found the hit compound **153** by fragment-based screening and further optimized to afford CPI-637 (**154**) [189] (Fig. 10c). Co-crystal structure data reveal that **154** reproduces key interactions between **153** and CBP bromodomain, including the canonical/water-mediated hydrogen bonds formed by lactam with Asn1168 and Tyr1125 (Supplementary Fig. 1f). Compound **154** is more than 700-fold more selective for CBP than BET proteins and is able to effectively inhibit MYC expression (EC_50_ = 0.60 μM). Almost simultaneously, the same group developed a new series of inhibitors characterized by a piperidino-pyrazole, including GNE-272 (**155**) [190], GNE-781 (**156**) [191], and GNE-207 (**157**) [192] (Fig. 10c). These compounds demonstrate potent inhibitory activity and extremely high selectivity towards CBP, with **156** being 5425-fold more selective than BRD4(1). Different from the “LPF shelf” (Leu1109, Pro1110, and Phe1111) in CBP, the spatial prominence of Trp81 from the “WPF shelf” in BRD4 forces the rotation of THQ, resulting in the formation of van der Waals interaction between the phenyl moiety and Leu92 and the exposure of *N*-methylpyrazole to the solvent. The spatially perfect complementarity of **156** and CBP maybe explain such ultra-high selectivity (Fig. 10a-b). In vivo*,* these compounds exhibited appreciable PK properties and significant AML growth inhibition.

By employing 1-(1*H*-indol-1-yl) ethanone as a scaffold, Xu and colleagues prepared a variety of innovative anti-prostate cancer CBP/p300 inhibitors (Fig. 10c), of which the representative compounds are **158** to **161.** Y08175 (**158**) displays a twofold superior CBP binding than that of **149**, with an IC_50_ of 37 nM, however this compound was shown ineffective in cellular assays [193]. The methyl ester form **159** has improved cellular permeability, and significantly inhibited the growth of prostate cancer cell lines LNcap and 22Rv1. Replacing its backbone with a 1-(medium nitrogen-3-yl) ethanone yielded Y08197 (**160**), which is also a highly selective and effective CBP/p300 inhibitor capable of suppressing the expression of AR-regulated genes (*PSA*, *KLK2* and *TMPRSS2*) and oncogenes (*MYC* and *ERG*) [194]. This compound significantly inhibits the growth, proliferation and migration of a variety of prostate cancer cell lines. Further optimization was performed to afford compound Y08284 (**161**)**,** which exhibits high selectivity for CBP/p300 with a significantly improved PK profile (t_1/2_ 21.23 min, Cl_int_ 81.87 mL/min/kg in human liver microsomes). In vivo, oral administration of 40 mg/kg of **161** five times a week for 14 continuous days was shown to dramatically inhibit prostate cancer tumor growth, with a TGI of 88% [195]. Through in silico screening and crystal-based structure optimization, Luo’s lab developed inhibitors DC-CPin711 (**162**) and DC-CPin734 (**163**) [196, 197] (Fig. 10c). Both inhibit the proliferation of various AML cell lines, especially MV4-11, with IC_50_ values of 1.2 μM and 0.55 μM. The 4-acyl pyrrole derivative XDM6 (or XDM-CBP, **164**) features a remarkable ligand efficiency and high selectivity, being capable of inhibiting the proliferation of numerous solid tumors and hematological malignancy cell lines, which confirms its feasibility and the potential of utilizing the 4-acyl pyrrole as a starting point for the development of non-BET inhibitors [198]. In addition, Christopher J et al. employed **156** as the warhead to engineer the CBP/p300 degrader dCBP-1 (**165**), capable of hijacking the ligase CRBN. Compound **165** can almost completely degrade CBP and p300 at a concentrations as low as 10 nM in a MM cell line (MM1S) [199].

### PCAF/GCN5 inhibitors

Numerous reports have documented the necessity to develop inhibitors of PCAF/GCN5, which are implicated in a variety of diseases, such as cancer, AIDS and inflammation. The BD of PCAF was confirmed to bind to acetylated K50 (K50Ac) and motivate Tat transactivation, ultimately leading to HIV transcription [200]. Zhou et al. obtained the first small molecule inhibitor NP1 (**166**, IC_50_ = 1.6 μM) of PCAF via NMR screening [201] (Fig. 10d). Through selectively inhibiting BD in PCAF, **166** is able to effectively block the binding of Tat-K50Ac and PCAF. Starting from a pyridazinone hit, GSK successfully developed GSK4027 (**167**), a highly selective and efficient chemical probe for PCAF/GCN5 [202]. Subsequently, **167** was employed as the ligand and tethered to thalidomide (Fig. 10d), affording a PCAF-targeting PROTAC, GSK983 (**168**, PCAF DC_50_ = 1.5 nM, GCN5 DC_50_ = 3.0 nM). Compound **168** was shown capable of effectively attenuating LPS-induced stimulation of macrophages and dendritic cells and reducing production of inflammatory cytokines [203]. In 2016, L-Moses (**169**, K_d_ = 126 nM) was reported as a highly potent, selective and cell-active chemical probe derived from triazolopthalazine to target PCAF [204]. This compound **169** shows good cell permeability and metabolic stability in in vitro assays. The co-crystal structure of **169** with *pf*GCN5 (BD from *Plasmodium falciparum*, 64% homology with PCAF BD) reveals that compound **169** is located in the KAc recognition pocket by forming a salt bridge with Glu1389, a π-π stacking with Trp1379/Tyr1442 and a water-mediated hydrogen bond with Asn1436 (Supplementary Fig. 1g). This study provides an outstanding basis for the design and development of subsequent PCAF inhibitors. Later, Yang et al. reported a novel and high selectivity PCAF inhibitor **170** (IC_50_ = 7 nM) with higher activity than **169** (IC_50_ = 36 nM, HTRF), which displayed favorable PK properties and cellular activity in HEK293T cells [205].

### TAF1 inhibitors

**Fig. 11:**
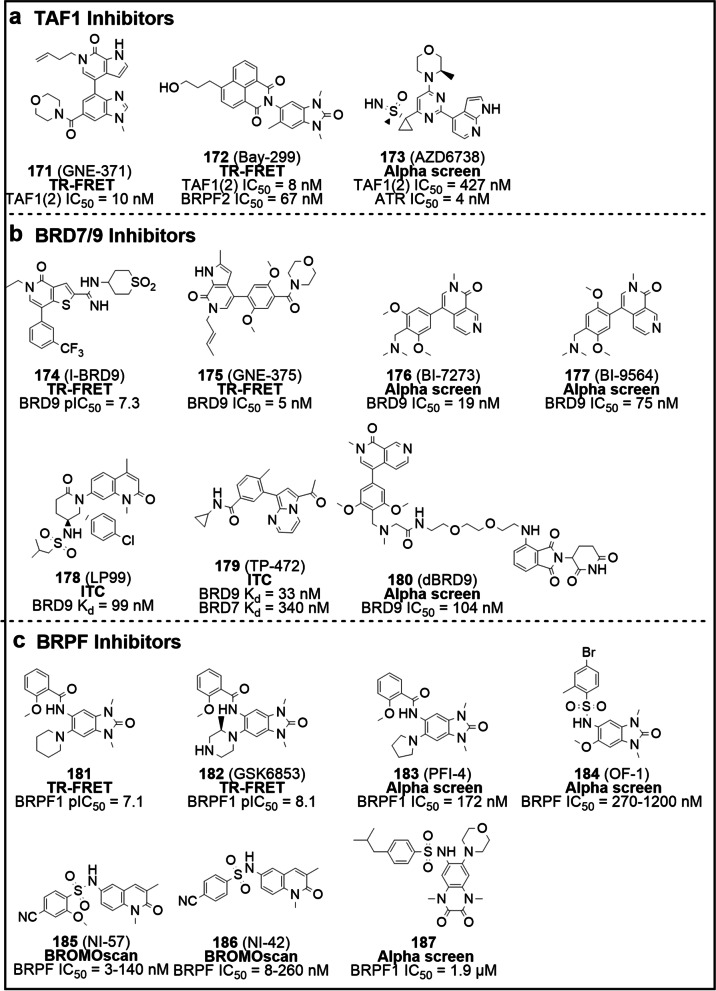
TAF1, BRD7/9, and BRPF inhibitors. **a** Chemical structures of TAF1 inhibitors **171** − **173**. **b** Chemical structures of BRD7/9 inhibitors **174** − **180**. **c** Chemical structures of BRPF inhibitors **181** − **187**. The activity test methods are bolded

Although the specific function of the two separate bromodomains BD1 and BD2 of transcription initiation factor TFIID subunit 1 (TAF1) remains ambiguous, they are recognized as potential targets for tumor therapy [206]. GNE-371 (**171**, IC_50_ = 10 nM), a potent inhibitor of TAF1(2), was reported by Genentech and Constellation (Fig. 11a). The 1-butenyl group on the pyrrolopyridone is the key moiety that can effectively improve the potency and selectivity of the compound by rearranging and stabilizing a conserved water network in the binding site [207]. It has been reported that a combination of TAF1 and BRD4 inhibitors offered synergistic anti-proliferative effects in tumor cells. A target engagement assay confirmed that **171** was able to increase the sensitivity of H23 lung cancer cells to **171** even at concentrations as low as 100 nM, suggesting that **171** is an efficient chemical probe useful for the exploration of the biological function of TAF1(2). Bayer synthesized a series of benzoisoquinolinedione derivatives based on HTS to obtain a novel BRPF2/TAF1 dual-target inhibitor (Fig. 11a), Bay-299 (**172**, TAF1(2) IC_50_ = 8 nM, BRPF2 IC_50_ = 67 nM). In the BROMOscan panel and in vitro studies, compound **172** displayed high selectivity and favorable PK properties, which indicates that this compound is a promising chemical probe to study BRPF2 and TAF1(2). In a separate report in 2021, compound **172** was shown capable of inducing MV4-11 and NB4 death in human AML cell lines via the RIKP1 signaling pathway, which reveals the potential of **172** to treat AML [208, 209]. Moreover, Schönbrunn et al. recently reported that ATR kinase inhibitor AZD6738 (**173**, Fig. 11a), which has advanced to clinical trials, inhibited TAF1(2) at a submicromolar level and was a potent dual-target TAF1/ATR inhibitor (TAF1(2) IC_50_ = 427 nM, ATR IC_50_ = 4 nM) [210]. Crystallographic and small-angle X-ray scattering studies reveals that when **173** binds with TAF1-T (containing BD1 and BD2), the morphology of TAF1-T changes from an open to a closed conformation. Therefore, compound **173** can not only inhibit the recognition function of BDs, but also affects transcription by interfering with the proper assembly and localization of TFIID in the preinitiation complex. These findings provide new insights into the development of TAF1 inhibitors.

### BRD7/9 inhibitors

Both BRD7 and BRD9 contain a unique BD, and the structural similarity and biological homology of the two individual BDs pose obstacles to the exploitation of selective inhibitors and chemical probes for BRD7 or BRD9. I-BRD9 (**174**) was the first selective BRD9 inhibitor discovered by GSK, with more than 700-fold and 200-fold selectivity over BET family proteins and BRD7, respectively [211] (Fig. 11b). With strong binding to BRD9 (pIC_50_ = 7.3), 10 μM of compound **174** markedly down-regulates genes (CLEC1, DUSP6, FES, and SAMSN1) related to cancer and immune pathways. Following this, Genentech and Constellation Pharmaceuticals developed a novel BRD9 inhibitor GNE-375 (**175**) based on a pyrrolopyridone core via structure-based drug design [212] (Fig. 11b). This compound fills the BRD9 with the 2-methoxy group occupying the accessible pocket adjacent to the lipophilic shelf, which is not tolerated in BRD4. Compound **175** displays high potency against BRD9 (IC_50_ = 5 nM), but practically no activity against 236 cell lines. Unexpectedly, **175** kills > 60% of PC9 cells while in combination with erlotinib and can down-regulate the ALDH1A1 gene associated with drug resistance, suggesting its potential in addressing tumor resistance. Boehringer Ingelheim developed two potent BRD9 inhibitors BI-7273 (**176**) and BI-9564 (**177**) by fragment-based and virtual screening [213] (Fig. 11b). Data from the literature demonstrate that both inhibitors have high affinity and strong inhibitory activity against BRD9, with desirable PK properties such as moderate to high solubility, metabolic stability, permeability and no CYP450 inhibition. These properties are beneficial for the subsequent elucidation of the biological role of BRD9. LP99 (**178**), the first selective BRD7/9 inhibitor developed by Oxford, exhibits anti-inflammatory potential by inhibiting LPS-stimulated IL-6 secretion in human monocytic leukemia cells [214]. Another probe, TP-472 (**179**), employed pyrrolo[1,2-a] pyrimidine as its structural skeleton (Fig. 11b), and showed high efficacy against BRD7/9. More recently, compound **179** was observed to inhibit melanoma cell growth in vitro and in vivo by down-regulating several extracellular matrix proteins and up-regulating pro-apoptotic genes [215]. Bradner et al. designed the first BRD9-targeting PROTAC dBRD9 (**180**) by linking a selective BRD9 inhibitor to the CRBN ligand (pomalidomide) via a PEG linker [216] (Fig. 11b). Compound **180** retains selective dimerization with BRD9, effectively inducing the degradation of BRD9 without noticeable off-target activity on BRD4 or BRD7. In human leukemia cell lines, the anti-proliferative efficacy of **180** was 10 to 100 times that of its parental inhibitors (**174** and **176**). This finding underlines the feasibility of developing non-BET degraders and the critical role of dBRD9 as a tool to explore the BRD9 biology or as a lead compound against leukemia.

### BRPF inhibitors

The multi-domain nature of bromodomain- and PHD finger-containing protein (BRPF), and the fact that they are usually located in protein complexes, lead to elusive functions of their BDs. Thus, targeted and efficient chemical probes against BRPF are needed. GSK reported compound **181**, a selective BRPF1 probe on the basis of benzimidazolone [217] (Fig. 11c). Compound **181** displays excellent binding activity towards BRPF1 (pIC_50_ = 7.1), exceeding BET and BRPF2/3 by 100 to 1000 fold**.** Structurally, Phe714 (Ile146 in BRD4) and Pro658 (Ser592 in BRPF2, Asn619 in BRPF3) residues in BRPF1 contribute to the selectivity of the probe (Fig. 11c). Structural optimization was further performed to obtain GSK6853 (**182**), featuring enhanced selectivity and activity, which is a suitable probe for cellular and in vivo studies [218] (Supplementary Fig. 1h). Furthermore, SGC, in collaboration with several pharmaceutical and academic institutions, conducts ongoing R&D on BRPF inhibitors and discovered compounds **183–186** [219–221] (Fig. 11c), including selective BRPF1 inhibitor PFI-4 (**183**) and pan-BRPF inhibitors OF-1 (**184**), NI-57 (**185**) and NI-42 (**186**). For the first time, preliminary findings suggest that BRPF is significantly implicated in cancer (AML), inflammation, and osteoclastogenesis, laying a firm foundation for exploring the biological functions and druggability of BRPF. The 2,3-dioxo-quinoxaline derivative compound **187** exhibits micromolar level of inhibitory activity against BRPF1 (IC_50_ = 1.9 μM) [222]. In BRPF2 and BRD4, the absence of critical van der Waals contacts with Ser592 (Pro658 in BRPF1), the spatial collision of the morpholine moiety with Trp81, and the smaller binding pocket resulted in the ineffectiveness of compound **187** for these two proteins.

### ATAD2 inhibitors

**Fig. 12:**
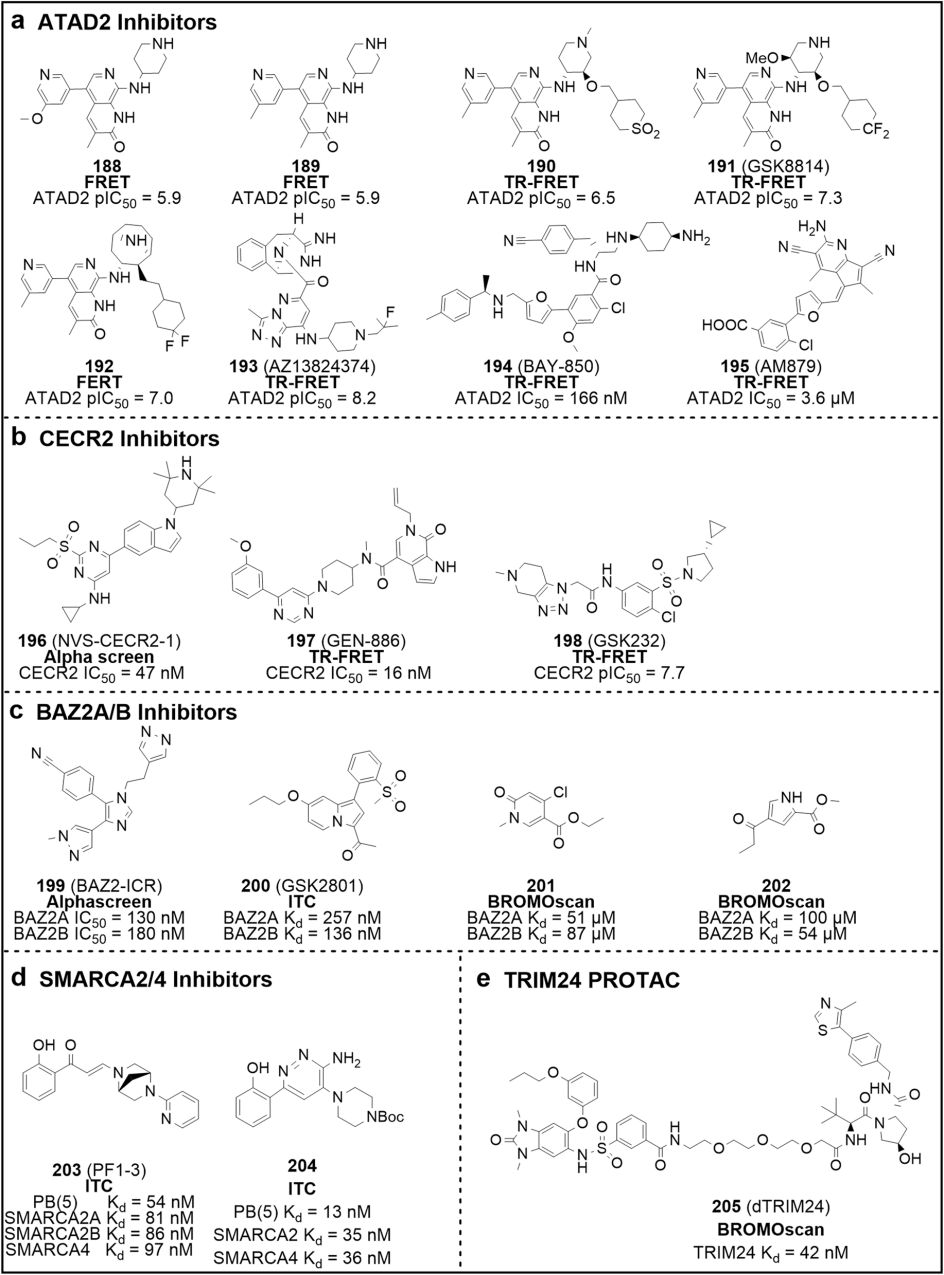
Other non-BET Inhibitors inhibitors. **a** Chemical structures of ATAD2 inhibitors **188** – **195**. **b** Chemical structures of CECR2 inhibitors **196** – **198**. **c** Chemical structures of BAZ2A/B inhibitors **199** – **202**. **d** Chemical structures of SMARCA2 /4 inhibitors **203–204**. **e** PROTAC of TRIM24. The activity test methods are bolded

Given the growing evidence that ATPase family AAA domain containing 2 (ATAD2) is implicated in various cancers and might be a marker of poor prognosis, chemical probes and inhibitors targeting this protein are gradually attracting the attention of investigators [223–226]. Starting with 3-methylquinolin-2(1*H*)-one, GSK developed micromolar inhibitors **188** and **189** based on the difference of binding pockets in the BD of ATAD2 and the specific acid cluster consisting of Asp1066, Asp1068, and Asp1071 [227] (Fig. 12a). Though the selectivity of these inhibitors is not satisfactory, the accessibility of ATAD2 inhibition with small molecules was evidenced. Further optimization yielded cell-permeable probe **190**, with sub-100 nM potency and a 100-fold selectivity over BET [228]. Chemical probes GSK8814 (**191**) and **192** were prepared using CF_2_ as the bio-isostere of sulfone [229, 230]. The former is the first low nanomolar and cell-permeable ATAD2 probe, while the latter uses tropane to restrict conformation. With the CF_2_ moiety exhibiting favorable electrostatic complementarity to the Arg1077 and “RVF shelf” (Arg1007, Val1008 and Phe1009) in ATAD2 (Supplementary Fig. 1i), both probes are highly selective. Recently, AZ13824374 (**193**) was discovered as a selective and potent ATAD2 inhibitor through HTS. This inhibitor was capable of reducing colony formation in a range of breast cancer models [231]. Furthermore, BAY-850 (**194**) and AM879 (**195**) were uncovered via a DNA-encoded library and structure-based virtual screening, respectively [232, 233]. Compound **194** can trigger BD dimerization, thus blocking KAc binding, while compound **195** can selectively inhibit ATAD2 and effectively induce autophagy through the PI3K-AKT-mTOR pathway in MDA-MB-231 cells.

### Other non-BET inhibitors

There are many non-BET inhibitors that have been reported, although their targeting BDs may have been initially regarded as having negligible likelihood of drug success.

As for cat eye syndrome critical region protein 2 (CECR2), NVS-CECR2-1 (**196**) was the first selective, potent and low-solubility CECR2 probe, co-developed by SGC and Novartis [234]. Followed by probes GEN-886 (**197**) and GSK232 (**198**), revealing the interaction mode with CECR2, their effects still need to be addressed [235, 236] (Fig. 12b).

BAZ2A/B was once considered unsuitable as a drug target due to the open and shallow binding site it possesses, until the chemical probe BAZ2-ICR (**199**) was discovered (Fig. 12c), which was typified by an internal π-stacking arrangement [237]. GSK2801 (**200**), a canonical acetyl lysine mimic, can displace BAZ2A from chromatin with appreciable PK properties [238]. Regrettably, after the discovery of active fragments **201** and **202** via in silico screening by Caflflisch et al. virtually no BAZ2A/B inhibitor has been reported [239].

For canonical family VIII bromodomains SMARCA2, SMARCA4 and PB1, both PF1-3 (**203**) and compound **204** were termed as SMARCA/PB1 chemical probes (Fig. 12d), and were employed to identify the roles of such proteins in adipogenesis [240–242].

Although TRIM24 is involved in a variety of cancers, selective inhibition of BD alone may not provide disease therapeutic benefit due to its multi-domain [243–246]. dTRIM24 (**205**) was the first selective PROTAC of TRIM24 depending on VHL [247] (Fig. 12e). Differential protein expression and cell cycle arrest resulting from TRIM24 depletion caused by **205** demonstrate the role of this enzyme in acute leukemias. This study suggests that degraders could also serve as powerful tools to probe the functions of candidate proteins, highlighting the advantage of holistic degradation of multi-domain proteins over selective inhibition of a single domain. Accordingly, it will be necessary to guarantee the high selectivity of PROTACs to avoid bias due to off-target effects.

## Perspective and conclusion

BCPs engage in histone modification, chromatin remodeling and transcription factors recruitment through recognition of KAc residues, while playing key roles in various physiological or pathological processes [5, 22, 248]. Since BD is the specific recognition site for KAc, waves of small molecule inhibitors and degraders have been disclosed over the past decade, especially BET-targeting inhibitors and PROTACs [34–37]. The current development of inhibitors is mainly done by simulating the mode of BD recognition KAc. Inhibitors fill the recognition pocket of KAc by forming hydrogen bonds with the conserved asparagine residue to produce biological effects. The strength of the interaction with residues and the degree of matching to the pocket determine the biological activity and selectivity of the inhibitors. Therefore, interactions with other amino acid residues are often constructed to enhance the activity of the drug, including the formation of additional hydrogen bond with Tyr97 (I-BET762) and salt bridge with Lys91 (PLX51107). In addition, in order to improve the selectivity of the molecule for BD or protein, it is particularly important to construct a unique interaction with the pocket, and a molecular shape that fits the pocket. For example, GSK778 forms a unique hydrogen bonding interaction with Asp144 to improve the selectivity of the molecule for BD1, RVX-208 is highly complementary to the pocket in shape to improve the selectivity for BD2, and the high fitness of GNE-781 to the “LPF shelf” makes it a highly selective CBP/p300 inhibitor. As co-crystal structures and SAR continue to accumulate, inhibitors are emerging with increasing druggable potential, and many have progressed to clinical trials. However, none were successfully approved for marketing so far, and plenty of challenges remain to be addressed. In particular, dose-limiting toxicity (DLT) due to pan-inhibition (pan-BET inhibitor) or off-targets is an essential factor in the failure of clinical trials. As mentioned earlier, the most common adverse effects include fatigue, gastrointestinal disorders (vomiting, diarrhea), hyperbilirubinemia, anemia and reversible thrombocytopenia. Accordingly, the development of subsequent new generations of inhibitors mainly may focus on the identification of high-efficiency and highly selective small molecules (such as RVX-208 and ABBV-744), rather than the original pan-inhibitors (( +)-JQ1, OTX015).

For BET inhibitor resistance, Wnt/β-catenin signaling is a recognized alternative pathway to keep *MYC* unaltered or to rapidly restore* MYC* transcription, while hyperphosphorylated BRD4 owing to the reduction of protein phosphatase 2A (PP2A) activity also facilitates drug resistance through its stable binding to MED1 [249–251]. Alternatively, phosphorylated BRD4, which is induced by IL6/IL8-JAK2 signaling, can maintain its stability by interacting with deubiquitinase UCHL3 [252], or by increasing conjugation to chromatin, thereby reducing binding to inhibitors. Activation of the AMPK/ULK1 pathway associated with the induction of pro-survival autophagy is also implicated in the resistance of leukemia stem cells (LSCs) to JQ1 [253]. A pharmacological combination approach is still an effective strategy to overcome tumor resistance [254–256], including BET inhibitors co-administered with Wnt/β-catenin inhibitors, BCL-xL inhibitors (ABT-737), CK2 inhibitors (Silmitasertib) or JAK2 inhibitors (pacritinib). Additionally, integrating different pharmacophores into a single molecule to achieve multifunctional inhibitors is also a fashionable approach to drug combinations. For example, the BRD4/CK2 dual-target inhibitor **131** exhibits favorable inhibitory activity against the JQ1-resistant MDA-MB-231 cell line, while the BRD4/HDAC inhibitor **116** can counteract drug resistance in colorectal cancer cells by blocking the IL6-JAK-STAT pathway. Besides, for some tumors (e.g. malignant peripheral nerve sheath tumor), simultaneously inhibiting BET activity and reducing BET levels may lead to synthetic lethality. Thus, PROTACs therapy demonstrates its unique protein modulatory benefits.

The flourishing of BET inhibitors is paralleled by the widespread development and coverage of non-BET family chemical probes or inhibitors. Numerous high-quality chemical probes have been discovered to facilitate the validation of protein functions, although sometimes the inhibition of a single domain does not trigger any biological effect in multi-domain proteins. For multi-domain proteins (such as p300/CBP, TRIM24, and SMARCA2A/2B), depleting the entire POI by efficient and specific PROTACs is an alternative and useful approach to explore the sophisticated biological role of each protein. Although milestones have been achieved for non-BET proteins and their inhibitors, of which the first non-BET inhibitor CCS1477 has advanced to clinical trials, the physiological features of some non-BET proteins remain obscure, and therefore the development of potent, selective, cell-permeable non-BET probes is still challenging.

Collectively, this paper provides a detailed review of the biology and inhibitors of BCPs, in which the study of typical BDs is relatively well-established, despite limited knowledge of atypical BDs (ASH1L, MLL, BRWD1, etc.). For BET inhibitors, the major challenge being faced is to improve clinical efficacy and minimize DLT. For non-BET inhibitors, the development of high-quality ligands to elucidate the physiological function and pharmacological potential of proteins remains a top priority. With the accumulation of knowledge on the structure, function and ligand binding mechanism of BCPs, as well as trial and error and optimization throughout the drug discovery process, novel BD-selective inhibitors or degraders will eventually be used in clinical practice to treat cancerous and inflammatory diseases.

## Supplementary Information

**Additional file 1: Supplementary Fig. 1.** Crystal structures of inhibitors bound to BCPs. a PBD ID: 3MXF. b PBD ID: 3P5O. c PBD ID: 3ZYU. d PBD ID: 4UYF. e PBD ID: 4ZW1. f PBD ID: 5I8G. g PBD ID: 5TPX. h PBD ID: 5G4R. i PBD ID: 5LJ0. j PBD ID: 5JWM. In co-crystal structures, key residues are highlighted with green sticks, while ligands are blue. Hydrogen bonds are shown in red dashed lines, the salt bridges are in purple, the π-π stacking is in yellow, and the electrostatic interactions are in wheat.

## Data Availability

Not applicable.
