# Supplementary material for: Targeting bromodomain-containing proteins: research advances of drug discovery
Source: Mol Biomed. 2023 May 5;4:13. doi: 10.1186/s43556-023-00127-1 (PMC10159834; doi:10.1186/s43556-023-00127-1)
Supplement: Supplementary file 1 — Additional file 1: Supplementary Fig. 1. Crystal structures of inhibitors bound to BCPs. a PBD ID: 3MXF. b PBD ID: 3P5O. c PBD ID: 3ZYU. d PBD ID: 4UYF. e PBD ID: 4ZW1. f PBD ID: 5I8G. g PBD ID: 5TPX. h PBD ID: 5G4R. i PBD ID: 5LJ0. j PBD ID: 5JWM. In co-crystal structures, key residues are highlighted with green sticks, while ligands are blue. Hydrogen bonds are shown in red dashed lines, the salt bridges are in purple, the π-π stacking is in yellow, and the electrostatic interactions are in wheat. [file 43556_2023_127_MOESM1_ESM.doc]

Supplementary Information for

Targeting bromodomain-containing proteins: Research Advances of Drug Discovery

**Fig. S1.**

**Supplementary Fig. 1** Crystal structures of inhibitors bound to BCPs. **a** PBD ID: 3MXF. **b** PBD ID: 3P5O. **c** PBD ID: 3ZYU. **d** PBD ID: 4UYF. **e** PBD ID: 4ZW1. **f** PBD ID: 5I8G. **g** PBD ID: 5TPX. **h** PBD ID: 5G4R. **i** PBD ID: 5LJ0. **j** PBD ID: 5JWM. In co-crystal structures, key residues are highlighted with green sticks, while ligands are blue. Hydrogen bonds are shown in red dashed lines, the salt bridges are in purple, the π-π stacking is in yellow, and the electrostatic interactions are in wheat.
